# Systems analysis of long-term heat stress responses in the C_4_ grass *Setaria viridis*

**DOI:** 10.1093/plcell/koaf005

**Published:** 2025-01-08

**Authors:** Peng Zhang, Robert E Sharwood, Adam Carroll, Gonzalo M Estavillo, Susanne von Caemmerer, Robert T Furbank

**Affiliations:** Research School of Biology, The Australian National University, Canberra, ACT 2601, Australia; ARC Centre of Excellence for Translational Photosynthesis, The Australian National University, Canberra, ACT 2601, Australia; ARC Centre of Excellence for Translational Photosynthesis, The Australian National University, Canberra, ACT 2601, Australia; Hawkesbury Institute for the Environment, Western Sydney University, Richmond, NSW 2753, Australia; Research School of Biology, The Australian National University, Canberra, ACT 2601, Australia; Commonwealth Scientific and Research Organisation, Agriculture and Food, Black Mountain Canberra, ACT 2601, Australia; Research School of Biology, The Australian National University, Canberra, ACT 2601, Australia; ARC Centre of Excellence for Translational Photosynthesis, The Australian National University, Canberra, ACT 2601, Australia; Research School of Biology, The Australian National University, Canberra, ACT 2601, Australia; ARC Centre of Excellence for Translational Photosynthesis, The Australian National University, Canberra, ACT 2601, Australia

## Abstract

Many C_4_ plants are used as food and fodder crops and often display improved resource use efficiency compared to C_3_ plants. However, the response of C_4_ plants to future extreme conditions such as heatwaves is less understood. Here, *Setaria viridis*, an emerging C_4_ model grass, was grown under long-term high-temperature stress for 2 wk (42 °C, compared to 28 °C). This resulted in stunted growth, but surprisingly had little impact on leaf thickness, leaf area-based photosynthetic rates, and bundle sheath leakiness. Dark respiration rates increased, and there were major alterations in carbon and nitrogen metabolism in the heat-stressed plants. Abscisic acid and indole-3-acetic acid–amino acid conjugates accumulated in the heat-stressed plants, consistent with transcriptional changes. Leaf transcriptomics, proteomics, and metabolomics analyses were carried out and mapped onto the metabolic pathways of photosynthesis, respiration, carbon/nitrogen metabolism, and phytohormone biosynthesis and signaling. An in-depth analysis of correlations between transcripts and their corresponding proteins revealed strong differences between groups in the strengths and signs of correlations. Overall, many stress signaling pathways were upregulated, consistent with multiple signals leading to reduced plant growth. A systems-based model of the plant response to long-term heat stress is presented based on the oxidative stress, phytohormone, and sugar signaling pathways.

## Teaser

Long-term heat stress does not affect photosynthesis in the C_4_ grass *Setaria viridis* owing to an effective acclimation response of carbon assimilation, although it inhibits plant growth.

## Introduction

C_4_ photosynthesis is a biochemical CO_2_ concentrating mechanism where CO_2_ is first assimilated by phosphoenolpyruvate carboxylase (PEPC) to produce a 4-carbon compound, a C_4_ acid (Hatch and Slack 1966; Hatch 1987). This C_4_ acid diffuses into the bundle sheath (BS) cell where decarboxylation of the C_4_ acid releases the previously fixed CO_2_ in a cellular compartment surrounded by suberin that restricts leakage of CO_2_ (Danila et al. 2021). This creates a high local concentration of CO_2_ around Rubisco so that the oxygenation reaction is suppressed, thus improving efficiency of CO_2_ assimilation while minimizing photorespiration. Most C_4_ plants have evolved the 2-cell mechanism, where CO_2_ entering the mesophyll (M) cell is first converted to HCO_3_^−^ by carbonic anhydrase (CA), which is then assimilated into phosphoenolpyruvate (PEP) by PEPC to make oxaloacetate (OAA). Depending on the decarboxylation enzyme used, OAA is converted to malate or aspartate, which is then transferred to adjacent BS cells where it is decarboxylated to release CO_2_ at the site of Rubisco (Hatch 1987; Furbank 2011).

C_4_ photosynthesis is considered an adaptation to environments that promote high photorespiration (Sage et al. 2018). The early lineages of C_4_ plants are thought to have evolved 25 to 30 million years ago, coinciding with the decline of atmospheric CO_2_ concentration from about 1,000 ppm to well below present day levels (Sage 2001, 2004; Zachos et al. 2001). In areas of the world where temperatures are frequently high, and drought and/or salinity are common, plants experienced high photorespiratory flux and were under selective pressure to evolve mechanisms that allow more efficient carbon gain (Sage et al. 2018). Presently, over 60 independent lineages of C_4_ plants have been discovered and altogether contributing toward ∼25% of the terrestrial primary productivity (Edwards et al. 2010). Some of the economically important C_4_ crops include maize (*Zea mays*), sorghum (*Sorghum bicolor*), millet (*Setaria italica*), and sugarcane (*Saccharum officinarum*). Given that C_4_ plants are regarded as relatively tolerant of higher growth temperatures (Berry and Bjorkman 1980), it is not surprising that the majority of studies on longer term effects of high-temperature stress on photosynthesis, metabolism, growth, and yield have been carried out on C_3_ plants (Sgobba et al. 2015; Kask et al. 2016; Hütsch et al. 2019; Haider et al. 2021; He et al. 2022).

Plant heat stress response during vegetative growth is extremely complex. The rates of most enzymatic reactions are sensitive to temperature increases, and heat stress can directly impact on protein and membrane stability, causing changes in the rates of biological processes. Heat may also cause the production of reactive oxygen species (ROS), which may act as signals to halt/alter plant growth but may also cause oxidative damage to the plant tissues leading to senescence and death (Bhattacharjee 2005; Sharkey 2005; Suzuki and Mittler 2006). Heat stress can significantly decrease plant photosynthetic capacity by negatively impacting on both the light and dark reactions. The structure and function of photosystem (PS) II is particularly heat labile (Sharkey 2005). Severe heat stress (above 45 °C) has been shown to directly cause structural damage to PS II, including dissociation of the oxygen evolving complex (Papageorgiou and Murata 1995) and dissociation of the reaction center protein complex (Nash et al. 1985). Moderate heat stress did not appear to severely affect PS II structure but was shown to inhibit the repair of photodamaged PS II by inhibiting the de novo synthesis of D1 protein, causing disruption to linear electron transport (Takahashi et al. 2004; Sharkey 2005; Allakhverdiev et al. 2008).

At elevated temperatures, both C_3_ and C_4_ Rubisco have been shown to be subject to deactivation due to heat-labile nature of Rubisco activase (RCA) and the buildup of inhibitors that reduce Rubisco activity (Sharkey et al. 2001; Crafts-Brandner and Salvucci 2002; Salvucci and Crafts-Brandner 2004a). In addition, the thermal lability of other Calvin–Benson cycle enzymes may also contribute to reduced carbon fixation under heat (Berry and Bjorkman 1980; Allakhverdiev et al. 2008). Heat stress has been shown to inhibit photosynthesis in C_4_ crop species such as sugarcane (40 °C), maize (above 38 °C), and sorghum (40 °C) with carbon fixation appearing to be more sensitive than light harvesting and electron transport (Blum 1986; Crafts-Brandner and Salvucci 2002; Wahid 2007; Perdomo et al. 2017). Furthermore, short-term heat stress (4 h) at 40 °C has been shown to inhibit photosynthesis in *S. viridis* plants (Anderson et al. 2021).

In addition to the direct impact of heat on photosynthetic components, high temperature may affect carbon fixation by influencing stomatal conductance. Under prolonged heat stress, plants may initially upregulate transpiration for evaporative cooling of the leaves, particularly if vapor pressure deficit is high. If availability of soil water is limited, this increase in transpirational water loss may induce water stress in the heat-stressed plants. Under such circumstances, a range of drought-related responses will be induced including stomata closure, which can limit CO_2_ availability to the chloroplast and inhibit photosynthesis. Water stress has been frequently observed in plants experiencing high temperature (Tsukaguchi et al. 2003; Wahid and Close 2007).

The impact of heat stress on plant growth depends on the plant species and the developmental stage. During vegetative growth, heat stress may inhibit cell expansion by lowering the water potential, leading to reduction in cell and overall plant size (Bañon et al. 2004; Potters et al. 2007). The developmental pattern may also be changed by heat stress. Changes in stomata and trichome densities and number of xylem vessels in both shoots and roots have been observed in *Lotus* plants (Bañon et al. 2004). Heat stress may inhibit the expansion of the first internode, which was observed in sugarcane plants grown under high temperature (Ebrahim et al. 1998). These plants also exhibited increased tillering, early senescence, and reduced biomass accumulation (Ebrahim et al. 1998). The impact of high temperature on enzymes directly involved in plant cell wall expansion is not well understood. In many cases, the reduction in plant growth caused by heat is thought to be the result of photosynthesis inhibition and altered assimilate partitioning (Wahid et al. 2007).

Given the advancement of various ‘omics technologies, and the huge complexity of plant heat stress response, there is a clear need for experiments employing a systems approach, by integrating multiple omics platforms to gain a holistic understanding of plant heat stress response and tolerance mechanisms in plants carrying out C_4_ photosynthesis. An experimental system was established to explore the long-term heat stress response of *S. viridis* seedlings. Seedlings were initially established under normal growth conditions (28 °C day/20 °C night) for 2 wk, then subjected to a heat stress treatment by transferring to a high-temperature growth chamber (42 °C day/32 °C night) for another 2 wk until the completion of vegetative growth and the onset of flowering. We consider this heat stress treatment “long term” because the length of the treatment corresponds to approximately half of the vegetative growth phase. Physiological, biochemical, transcriptomics, proteomics, and metabolomics data were measured in control and heat-treated plants and compared to determine mechanisms of perception of the stress and the plant system response.

## Results

### Response of growth and photosynthesis to high temperature

**Figure 1.**
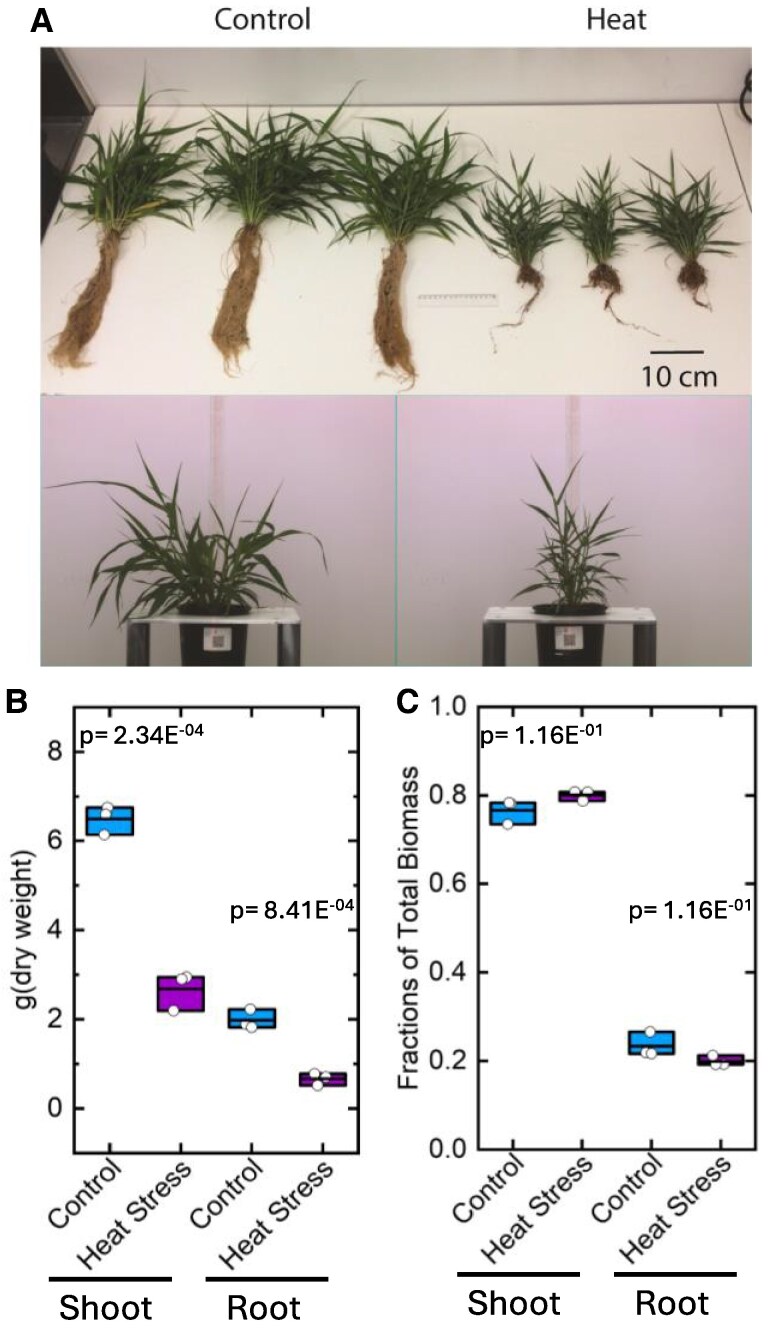
The growth and biomass accumulation of *S. viridis* in response to heat stress. **A)** Representative photos of plants grown at 28 °C day/22 °C night (Control) and 42 °C day/32 °C night (Thoreen et al. 2012). **B)** The shoot and root dry biomass and **C)** biomass allocation to the shoot or root as a fraction of total biomass in the control and heat-stressed plants. Data points are overlayed in the box plots with the straight line representing the mean with the lower and upper edge representing the interquartile range (25% to 75%), and *P* values were calculated from Student's *t* tests (2-tailed, assuming equal variance, *n* = 3).

Growth of *S. viridis* at 42 °C/32 °C day–night cycle resulted in a marked stunting of plant growth (Fig. 1A). Both the shoot and the root dry biomass were reduced by approximately 50% compared to the plants grown at 28 °C/22 °C, while the root to shoot ratio was largely unaffected (Fig. 1, B and C).

Anatomical studies showed the youngest fully expanded leaves were smaller in heat-stressed plants, but leaf thickness was not significantly different, while interveinal distance was slightly reduced (Supplementary Fig. S1).

**Figure 2.**
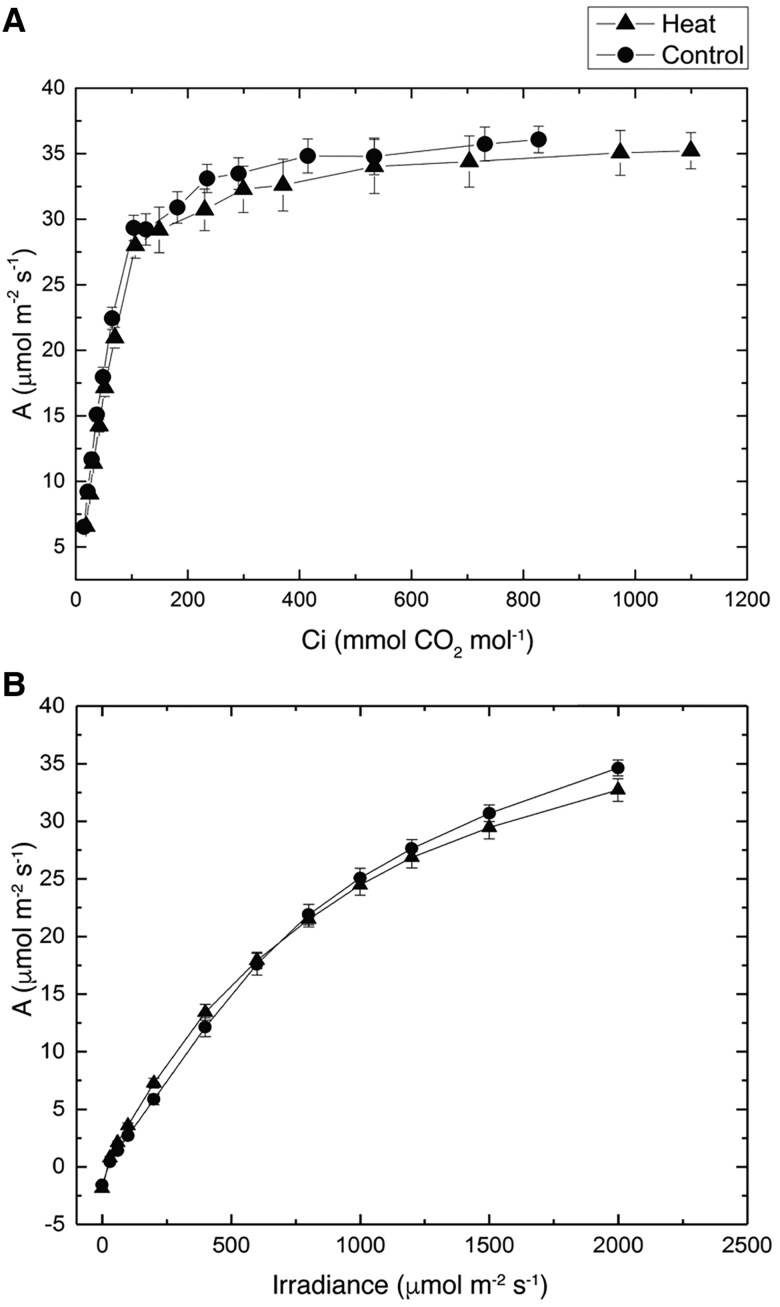
**A)** The CO_2_ assimilation rate (*A*) of the flag leaf in response to increasing concentrations of CO_2_, i.e. the *A*/Ci curve, of the control and heat-stressed plants. Ci represents leaf internal CO_2_ concentration. Measurements were taken at 25 °C and irradiance of 2,000 *μ*mol m^−2^ s^−1^. The initial slopes of the *A*/Ci curves for each replicate plant were calculated separately by fitting a linear regression line through the first 5 data points. The initial slope for the control plants was 0.34 ± 0.01 and 0.31 ± 0.01 for the heat-stressed plants (*P* = 0.18) (*N* = 4). **B)** The response of CO_2_ assimilation rate to changing light intensity in the control and heat-stressed plants. Measurements were performed at ambient CO_2_ concentration of 380 *μ*bar and 25 °C. Closed circles represent means of 4 biological replicates of plants grown at 28 °C day/22 °C night. Closed triangles represent means of 4 biological replicates of plants grown at 42 °C day/32 °C night. Student's *t* tests (2-tailed, assuming equal variance) were used for *P* value calculation (*n* = 4).

Despite the significant effects on plant growth and development, growth at high temperature had no significant impact on photosynthetic characteristics of young fully expanded flag leaves (Table 1 and Fig. 2). The maximum rates of photosynthesis on an area basis achieved at saturating CO_2_ partial pressure and high light measured at 25 °C for high-temperature-grown plants were not different from plants grown at control temperatures. Nevertheless, an increase in the thermal optimum of photosynthesis was observed (Table 1). Total Rubisco activity and activation status were similar between treatments; however, heat-stressed plants displayed significant increases in PEPC activity and the PEPC:Rubisco ratio. The response of photosynthetic rate to CO_2_ concentration changes at high light and to light level changes at ambient CO_2_ was indistinguishable from plants grown at control temperatures (Fig. 2). Similarly, the photosynthetic carbon assimilation response at varying temperatures did not differ between heat-stressed and nonstressed plants (Supplementary Fig. S2). Furthermore, BS leakiness (ϕ) determined from online stable isotope discrimination was unchanged across measurement temperatures, which indicated CCM function was not perturbed between treatments (Supplementary Fig. S2).

**Table 1.** Summary of biochemical characteristics of the leaf of *S. viridis* plants grown at 28 °C day/22 °C night or 42 °C day/32 °C night (*N* = 4 for each parameter)

	Growth conditions		*P* value (*t* test)
	28 °C/22 °C	42 °C/32 °C	
*A*, at 25 °C (*µ*mol m^−2^ s^−1^)	28.81 ± 3.53	23.55 ± 1.62	2.46E^−01^
*A*, at growth temperature	32.19 ± 3.50	30.09 ± 2.12	6.32E^−01^
*T*_opt_ for *A* (°C)	34.86 ± 0.70	37.25 ± 0.44	3.70E^−02^
*A*, at 45 °C (*µ*mol m^−2^ s^−1^)	17.30 ± 2.27	22.13 ± 2.10	1.69E^−01^
Rubisco activity (initial) (*µ*mol CO_2_ m^−2^ s^−1^)	16.23 ± 2.32	20.29 ± 3.07	3.51E^−01^
Rubisco activity (activated) (*µ*mol CO_2_ m^−2^ s^−1^)	22.61 ± 1.74	27.82 ± 1.00	6.02E^−02^
Rubisco activation %	71.86 ± 8.26	72.82 ± 10.55	9.46E^−01^
PEPC activity (*µ*mol CO_2_ m^−2^ s^−1^)	187.82 ± 6.96	301.44 ± 10.11	7.60E^−04^
PEPC:Rubisco	8.36 ± 0.36	10.84 ± 0.05	2.50E^−03^
Chlorophyll a + b (mmol m^−2^)	0.70 ± 0.03	0.68 ± 0.06	7.67E^−01^
Chlorophyll a/b	6.56 ± 0.26	5.29 ± 0.35	2.00E^−02^
C:N ratio	23.01 ± 1.61	14.79 ± 1.43	2.43E^−03^
Total protein content	5.90 ± 0.15	6.60 ± 0.13	6.11E^−03^

### Transcript, protein, and metabolite profiles

#### The C_4_ cycle

**Figure 3.**
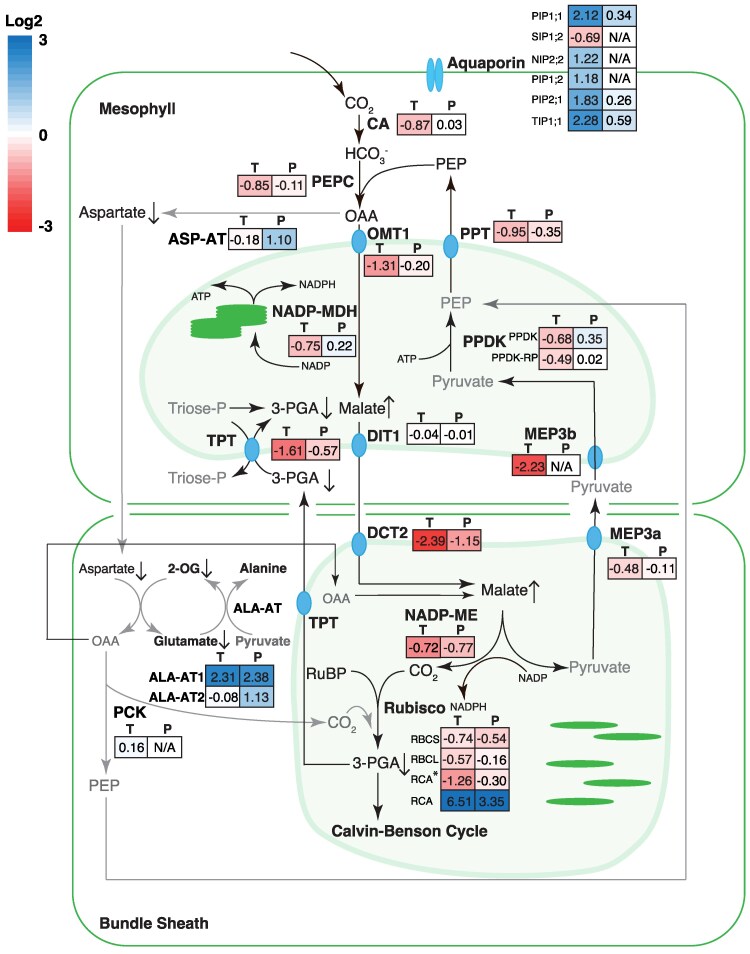
The response in expression of genes involved in the C_4_ pathway at the transcript and protein level, and the associated changes in metabolites to heat stress treatment. For each gene, the transcript and protein fold changes (heat vs control) in log_2_ scale are shown in colored boxes, where “T” and “P” designate transcript and protein levels, respectively. The color scale for transcript and protein log_2_ fold changes can be found on the top left corner. The arrows next to metabolite names indicate significant changes (*P* < 0.05) in their relative levels as detected by GC/MS. Metabolites not detected were shown in gray. Relative metabolite levels are shown in Supplementary Data Set 1. CA, carbonic anhydrase; PEPC, phosphoenolpyruvate carboxylase; ASP-AT, aspartate amino transferase; OMT1, oxoglutarate:malate antiporter; NADP-MDH, NADP-dependent malate dehydrogenase; PPDK, pyruvate orthophosphate dikinase; DIT1, dicarboxylate transporter 1; TPT, triose-phosphate translocator; NADP-ME, NADP-dependent malic enzyme; Rubisco, ribulose bisphosphate carboxylase/oxygenase; ALA-AT, alanine aminotransferase; PCK, phosphoenolpyruvate carboxykinase; DIT2, dicarboxylic transporter 2; MEP3a/3b, proton:pyruvate cotransporter 3a/3b; PPT, PEP/phosphate translocator; TPT, triose-phosphate translocator.

To further investigate the effect of long-term heat stress on the functioning of the C_4_ cycle, the expression of genes involved in the C_4_ pathway was examined using both RNA-seq and quantitative proteomics methods, coupled to metabolite measurements by GC/MS. Levels of mRNA transcript (T), protein (P), and metabolites in leaves of the control and heat-stressed plants are shown in the color-coded diagram in Fig. 3. Numbers in the text box next to enzyme abbreviation represent the ratio between heat-stressed versus control plants in log_2_ scale. Although transcript levels of core C_4_ cycle enzymes (CA; PEPC; NADP-MDH, NADP-malate dehydrogenase; RBCL, Rubisco large subunit; RBCS, Rubisco small subunit; and PPDK, pyruvate orthophosphate dikinase) were significantly lower under heat stress, in most cases, there were no significant reductions in the amount of proteins, except for NADP-ME and RBCS (Fig. 3). Similarly, the transcript levels of transporters for C_4_ pathway metabolites—oxoglutarate:malate antiporter (OMT1), dicarboxylate transporter 1 (DIT1), dicarboxylic transporter 2 (DIT2), proton:pyruvate cotransporter 3a/3b (MEP3a/3b), PEP/phosphate translocator (PPT), and triose-phosphate translocator (TPT) (OMT1, DIT1, DCT2/DIT2, MEP3a and b, PPT, TPT) (Weber and von Caemmerer 2010; Schlüter et al. 2016)—were significantly lower. A significant reduction at the protein level was only observed for the dicarboxylate transporter DCT/DIT2, which transports malate into the BS chloroplast for decarboxylation (Weissmann et al. 2016). There was a significant buildup of malate in the leaves of heat-stressed plants, although it is unclear in which cell type or subcellular compartment the malate accumulated.

Some NADP-ME type C_4_ plants such as *Z. mays* and *Flaveria bidentis* produce a significant amount of aspartate in the mesophyll cells as the C_4_ metabolites for translocation of carbon to the BS cells, where it can be converted to OAA and decarboxylated by PEP carboxykinase (PEPCK), or reduced to malate and decarboxylated by NADP-ME (Furbank 2011). In our experiment, the expression of aspartate aminotransferase (ASP-AT), which converts OAA into aspartate, was significantly upregulated at the protein level under heat stress but PEPCK transcript was undetectable (Fig. 3; Supplementary Table S1). Concordantly, the expression of 2 alanine aminotransferases (ALA-AT) was also upregulated significantly at the protein level. The levels of aspartate, 2-OG, and glutamate significantly reduced in the heat-stressed plants (Fig. 3). The heightened flux in the reaction pathway to producing PEP from aspartate suggests that under heat stress, aspartate is an important C_4_ acid of the C_4_ cycle, potentially allowing the products of PEP carboxylation to move to the BS cells under conditions where the mesophyll chloroplast stroma is becoming more oxidized and less able to reduce OAA to malate. Interestingly, we also observed a concurrent highly significant decrease in the level of beta-alanine (Supplementary Data Set 1), a metabolite long suspected (but so far not proven) to be derivable from aspartate in plants that has previously been observed to *increase* under heat stress in divergent species (Parthasarathy et al. 2019) and linked to differential tolerance against abiotic stresses, including heat stress, in plants (Cross et al. 2004; Fouad and Altpeter 2009; Glaubitz et al. 2017; Devnarain et al. 2019; Parthasarathy et al. 2019).

#### The Calvin–Benson–Bassham cycle

**Figure 4.**
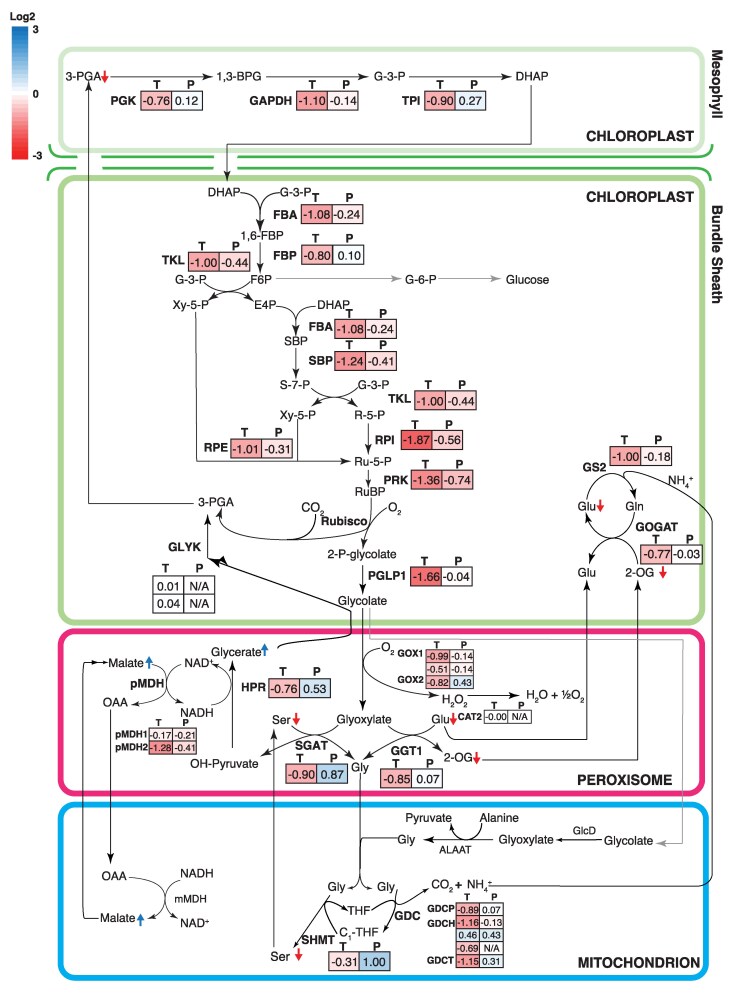
The response in expression of genes involved in the Benson–Calvin cycle and the photorespiratory cycle at both the transcript and protein level, and the associated changes in metabolites involved in the pathways to heat stress. The color codes of the diagram are the same as described in Fig. 3. The arrows (blue for increased and red for decreased) next to metabolite names indicate significant changes in their relative levels as detected by GC/MS. PGK, phosphoglycerate kinase; GAPDH, glyceraldehyde-3-phosphate dehydrogenase; TPI, triose-phosphate isomerase; FBA, fructose-bisphosphate aldolase; FBP, fructose bisphosphatase; TKL, transketolase; SBP, sedoheptulose-1,7-bisphosphatase; RPE, ribulose-phosphate3 epimerase; RPI, ribose-5-phosphate isomerase; PRK, phosphoribulose kinase; PGLP1, phosphoglycolate phosphatase1; GOX, glycolate oxidase; SGAT, serine:glyoxylate aminotransferase; GGT, glutamate:glyoxylate aminotransferase; SHMT, serine hydroxymethyltransferase; GDC, glycine decarboxylase; mMDH, mitochondrial malate dehydrogenase; pMDH, peroxisomal malate dehydrogenase; HPR, hydroxypyruvate reductase; CAT, catalase; GS, glutamine synthetase; Fd-GOGAT, ferredoxin-dependent glutamate synthase; GLYK, glycerate kinase.

The effect of heat stress on the expression of genes involved in the Calvin–Benson–Bassham (CBB) and the photorespiratory cycles was also examined. The complexity of the traffic of 3-C sugar phosphates between the mesophyll and BS cells of C_4_ plants (von Caemmerer and Furbank 2016) and the absence of cell-specific data in this study require some assumptions in interpreting data. The pathway diagram shown in Fig. 4 is based on cell-specific gene expression data in the 2 cell types (John et al. 2014) where each gene is placed in the location where it is predominantly expressed. All of the genes involved in the CBB cycle showed significant reduction at the transcript level in the heat-stressed plants compared to the control (Fig. 4). However, at the protein level, only phosphoribulokinase (PRK) reduced significantly at high temperature, while other enzymes were unchanged.

Notably, both transcript and protein levels of an isoform of RCA with a C-terminal extension (Supplementary Fig. S3) were strongly induced and almost exclusively expressed under heat stress (the transcript increased ∼90-fold and the protein increased ∼10-fold under heat treatment). It is known that RCA is a key heat-sensitive step in photosynthesis (Law and Crafts-Brandner 1999; Salvucci and Crafts-Brandner 2004b). The C-terminal tail has been suggested to play a role in Rubisco activation under heat stress in both wheat and maize, as a longer RCA isoform has been reported to be more heat stable (Crafts-Brandner and Salvucci 2002; Ristic et al. 2009).

#### The photorespiratory cycle

Although C_4_ plants have a largely diminished level of photorespiration, the genes involved in the photorespiratory cycle were still highly expressed in *S. viridis*, based on the mean RPKM of the photorespiratory genes relative to genes involved in the Benson–Calvin cycle (Supplementary Table S1). Although, at the transcript level, expression of phosphoglycolate phosphatase1 (PGLP1), glycolate oxidase (GOX), glutamate:glyoxylate aminotransferase 1 (GGT1), serine:glyoxylate aminotransferase (SGAT), hydroxypyruvate reductase (HPR), glycine decarboxylase (GDC subunits), peroxisomal malate dehydrogenase (pMDH2), ferredoxin-dependent glutamate synthase (Fd-GOGAT), and glutamine synthetase 2 (GS2) were reduced, the protein levels of SGAT, serine hydroxymethyltransferase (SHMT), and HPR significantly increased under heat stress (Fig. 4). Accompanying these changes was a significant reduction in the level of serine and a significant increase in the accumulation of glycerate (Fig. 4; Supplementary Fig. S5). This glycerate, produced in the photorespiratory cycle, needs to be converted back to 3-PGA to complete the cycle. It is uncertain whether changes in the level of glycerate kinase (GLYK) would have any effect on the accumulation of glycerate as GLYK protein was not detected. Metabolites involved in the NH_4_^+^ assimilation part of the photorespiratory cycle, including glutamate and 2-oxoglutarate, also showed significant reduction in the heat-stressed plants (Fig. 4).

### Expression of genes involved in chloroplast electron transport

**Figure 5.**
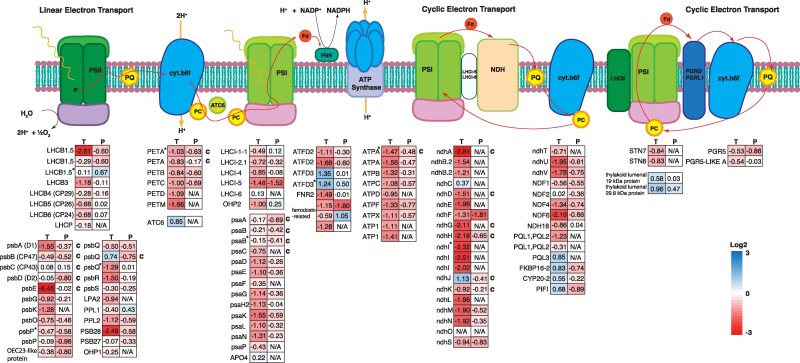
Expression of chloroplast electron transport genes under heat stress treatment. Schematics of protein complexes of the chloroplast electron transport chain located on the thylakoid membrane are drawn. Linear electron transport includes the PS II, Cytochrome b6f, PS I, and ATP synthase complexes. There are 2 possible modes of cyclic electron transport that involve PS I, NDH complex, or PGR5 protein. The expression level of the subunits of each protein complex is shown below the schematic diagram. Numbers shown are the log_2_-transformed fold changes between the heat-stressed plants and control plants. The left column shows the expression level of transcripts and the right column shows protein level. When multiple isoforms of the same subunit exist, an asterisk mark is used to designate the most highly expressed isoform. A letter “c” is written next to genes encoded by the chloroplast genome. The color scale can be found on the bottom right corner.

Chloroplast electron transport consists of both linear and cyclic electron transport. The M and BS cells host linear and cyclic electron transport chain components, although cyclic electron transport occurs primarily in the BS cells in NADP-ME type C_4_ plants (Fig. 5). The expression of genes in each protein complex in the electron transport chain was examined at the transcript and protein level. Transcripts encoding several components of the light harvesting complex (LHC) II and PS II were expressed at a lower level under heat stress. In addition, the protein levels for PS II extrinsic protein O, P, and Q (psbO, psbP, and psbQ), which are components of the oxygen evolving complex, also reduced significantly at the protein level (Fig. 5). All of the subunits of Cytochrome b6f complex exhibited significant reduction in mRNA levels, with Cytochrome f (PETA) and Rieske FeS protein (PETC) also reduced significantly at the protein levels (Fig. 5). Similarly, many genes encoding LHC I and PS I subunits showed significant decreases in expression at the transcript level, but changes in the corresponding proteins were mostly not significant. Subunits of ATP synthase all exhibited a similar magnitude of reduction by heat stress (except ATPD) at the transcript level, while protein abundance of ATP synthase subunits ATPB, ATPC, ATPF, and ATPX also decreased significantly. The NAD(P)H dehydrogenase-like complex (NDH complex), involved in cyclic electron transport activity (Johnson 2011), showed a pronounced reduction of all subunits at the transcript, and many of the subunits detected in the proteomics study were also significantly reduced. Expression of another protein involved in PSI cyclic electron transport, the proton gradient regulation 5 (PGR5) protein (Johnson 2011), was also reduced at both the transcript and protein level under high temperature.

Of note is the concerted reduction of expression of different subunits of the same complex. For example, expression of the subunits of Cytochrome b6f, ATP synthase, and NDH complex all showed similar levels of reduction at the transcript level. This suggests that the expression of these subunits of the same complex might be under the regulation of the same transcription regulon. Despite the global reduction in expression in the components of chloroplast electron transport chain, electron transport capacity was maintained in the heat-stressed plants (Fig. 2).

### Soluble sugars and starch levels

**Figure 6.**
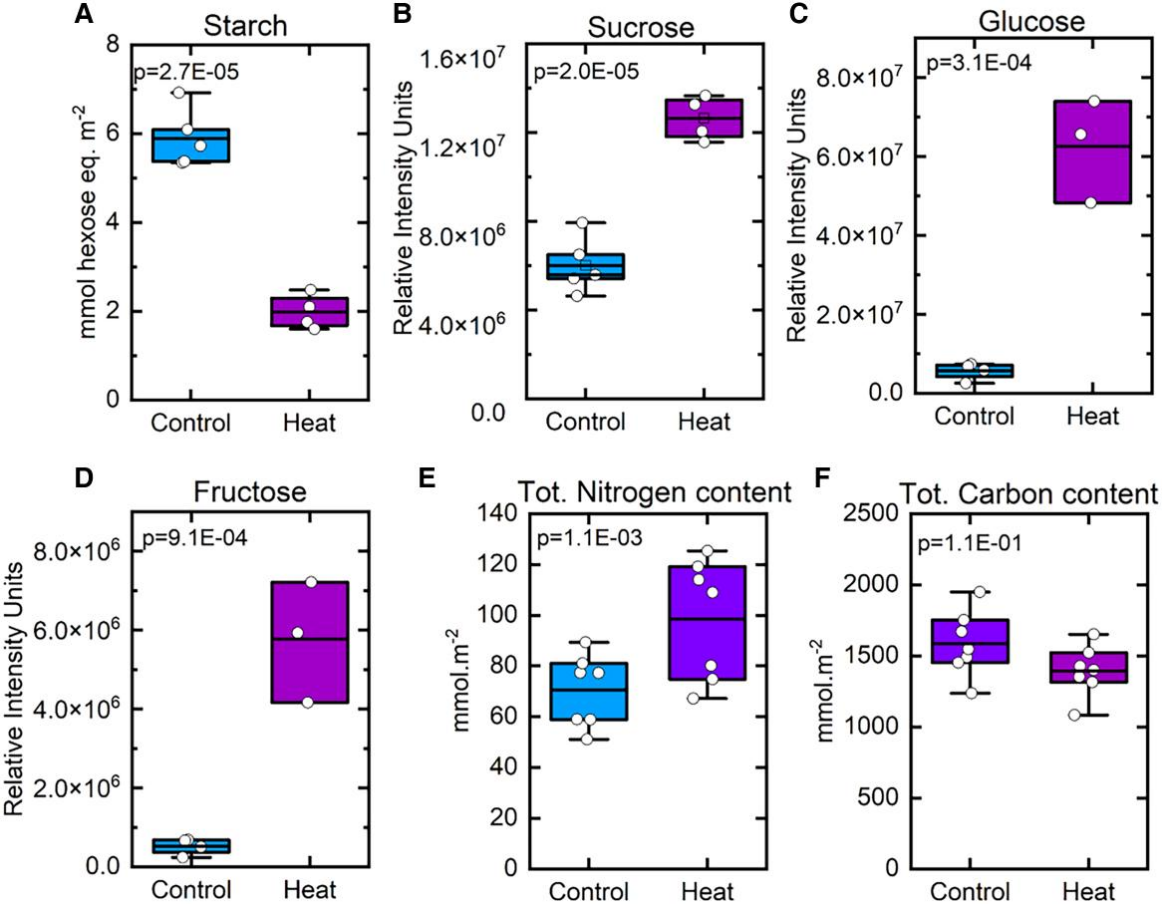
The levels of starch, soluble sugars (sucrose, glucose, and fructose), total nitrogen, and carbon contents in the control and heat-stressed plants are shown in box plots, with data obtained from measurements of 5 to 6 biological replicates. The levels of soluble sugars are shown as relative concentrations, in units of relative intensity, which represents the signal intensity of the derivatized compounds on a GC/MS. Data points are overlayed in the box plots with the straight line representing the mean with the lower and upper edge representing the interquartile range (25% to 75%), and *P* values were calculated from Student's *t* tests (2-tailed, assuming equal variance).

The leaf starch and sugar contents in 4-wk-old *S. viridis* plants grown at 28 °C (control) or 42 °C (heat-stressed) were measured in leaf samples collected at midday. On a leaf area basis, heat-stressed plants accumulated significantly smaller amounts of starch compared to the control counterparts (Fig. 6A). This result was further confirmed by TEM images of the leaves, showing reduced accumulation of starch granules inside chloroplasts in the heat-stressed plants compared to the control (Supplementary Fig. S4).

**Figure 7.**
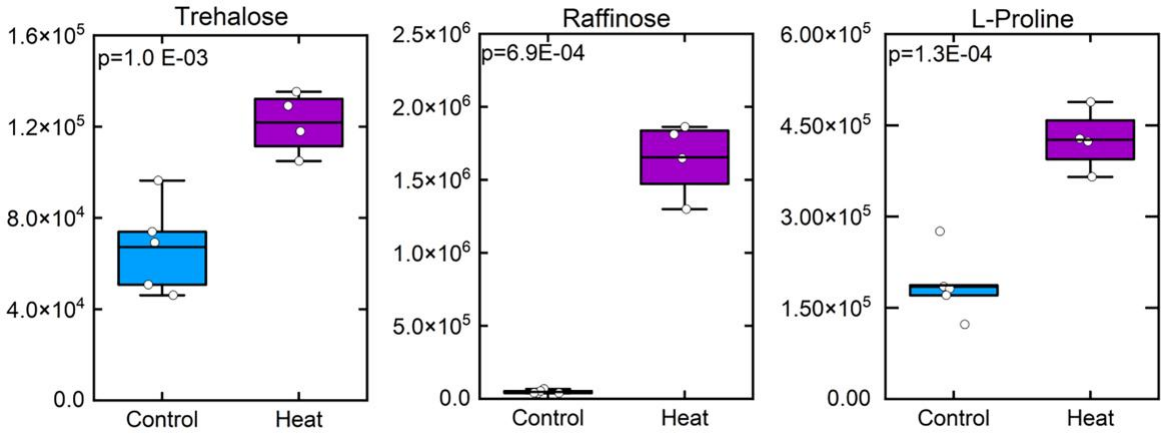
The relative levels of trehalose, raffinose, and proline in the control and heat-stressed plants, as determined by GC/MS metabolomics, are shown in box plots. Values represent the integrated peak area of the *m*/*z* channel used to quantify each analyte after normalization to the peak area of the ribitol internal standard in the same sample. Peak area of the quantifier ion for each metabolite, in arbitrary signal intensity unit. These values are only comparable between measurements of the same metabolites, not between different metabolites. Compounds significantly increased (*P* < 0.05) are indicated as determined by the Student's *t* test (2-tailed, assuming equal variance; *n* = 4 or 5). Data points are overlayed in the box plots with the straight line representing the mean with the lower and upper edge representing the interquartile range (25% to 75%).

The relative levels of leaf-soluble sugars were measured by GC/MS metabolomics and are shown in units of relative intensity in Fig. 6, B to D. The fold changes of all the metabolites measured by GC/MS and their *P* values can be found in Supplementary Fig. S5. The heat-stressed plants accumulated significantly higher levels of sucrose, glucose, and fructose in leaves (Fig. 6, B to D). The level of glucose-6-phosphate did not change significantly in the heat-stressed plants compared to the controls, and there was only a small but significant increase in the level of fructose-6-phosphate (Supplementary Fig. S5). The levels of trehalose, raffinose, and proline increased strongly in the heat-stressed plants (Fig. 7). In addition, a number of other soluble sugars (including cellobiose, gentiobiose, maltose, arabinose, ribose, xylulose, and mannose) and sugar alcohols (including D-threitol, erythritol, sorbitol, and xylitol) also showed significantly increased levels in the heat-stressed plants (Supplementary Fig. S5).

Interestingly, while leaf protein levels increased only slightly in heat-stressed leaves, nitrogen content (Fig. 6, E and F) increased and the carbon to nitrogen ratio of the tissue under heat stress declined by almost 50% (Table 1).

Taken together, these results showed the significant accumulation of a wide range of soluble sugars and their derivatives in the heat-stressed *S. viridis* plants, whereas the accumulation of transitory starch significantly reduced under heat.

### Expression of genes encoding enzymes of carbohydrate metabolism

**Figure 8.**
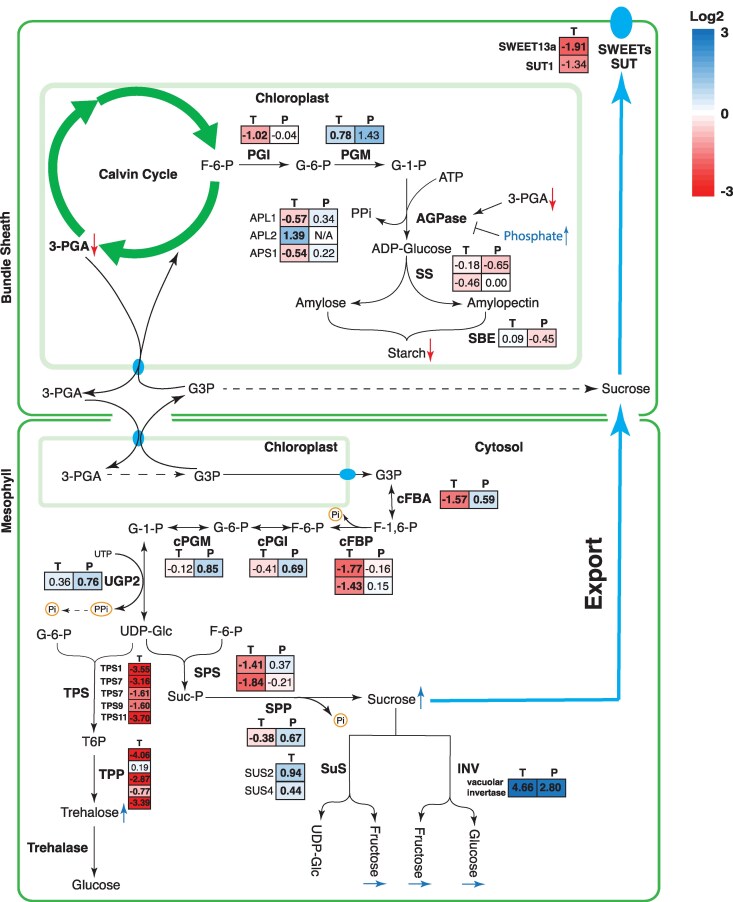
Changes in the expression of genes encoding for proteins involved in starch synthesis, sucrose synthesis/breakdown, and trehalose metabolism. For each gene, the transcript and protein fold changes (heat vs control) in log_2_ scale are shown in colored boxes, where “T” designates the change in transcript level and “P” designates protein level. The color scale can be found on the top right corner. The arrows (blue for increased and red for decreased) next to metabolite names indicate significant changes in their relative levels as detected by GC/MS. Relative metabolite levels are shown in Supplementary Data Set 1. Metabolites that were not measured are shown in gray. Starch was measured by the Megazyme Starch Assay Kit.

Given the large reprogramming of sugar and starch metabolism during heat stress shown in Figs. 6 and 7, expression of genes encoding enzymes in starch and sugar biosynthesis were examined (Fig. 8). For simplicity of the diagram, Fig. 8 uses the proposed cell localization of carbohydrate metabolism for maize (Lunn and Furbank 1999), with starch synthesis only shown inside the BS cell and sucrose synthesis/breakdown only shown inside the M cell.

The genes involved in starch synthesis, AGPase large subunit (APL1) and small subunits (APS1), Starch Synthase (SS), and Starch Branching Enzyme (SBE) did not show significant changes at the level of protein abundance in the heat-stressed plants, although the transcript levels of APL1 and APS1 significantly decreased (Fig. 8).

The genes involved in sucrose synthesis, including cytosolic Fructose-Bisphosphate Aldolase (cFBA), cytosolic Glucose-6-phosphate Isomerase (cPGI), cytosolic Phosphoglucomutase (cPGM), UDP Glucose Pyrophosphorylase (UGP2), and Sucrose Phosphate Phosphatase (SPP), all increased significantly in terms of protein abundance. However, the transcript abundance of cFBA, cFBP, and Sucrose Phosphate Synthase (SPS) significantly decreased under heat (Fig. 8). The expression of sucrose synthase (SUS) and vacuolar invertase (INV) significantly increased under heat, and the protein abundance for vacuolar INV also showed a significant increase. Metabolomics data (Fig. 7) showed a significant increase in sucrose, glucose, and fructose levels in the heat-stressed plants consistent with upregulation of sucrose synthesis and its breakdown into hexoses under heat. The hexose sugars might be accumulating inside the vacuole as vacuolar INV was significantly upregulated.

The synthesis of trehalose-6-phosphate (T6P) has been shown to be closely correlated with the amount of sucrose in the cells (Paul 2007; Figueroa and Lunn 2016). In the current experiment, we could not measure the level of T6P, but the level of trehalose significantly increased in the heat-stressed plants. However, transcript levels for various Trehalose-6-phosphate Synthase (TPS) and Trehalose-6-phosphate Phosphatase (TPP) isoforms have all decreased significantly and the protein products were not detected in this experiment (Fig. 8).

In concert with the significant accumulation of raffinose (∼35-fold increase compared to control) and galactinol (∼4-fold increase) in response to high temperature, the expression of galactinol synthase 1 (GolS1), raffinose synthase, and stachyose synthase were significantly upregulated at the transcript level (Supplementary Fig. S6). The expression of GolS1 and 2 and stachyose synthase are preferentially localized to M cells while 1 raffinose synthase isoform preferentially localizes to BS cells and the other 2 isoforms do not show cell type preference in the published cell-specific expression data sets of John et al. (2014).

### Changes in hormones under heat

**Figure 9.**
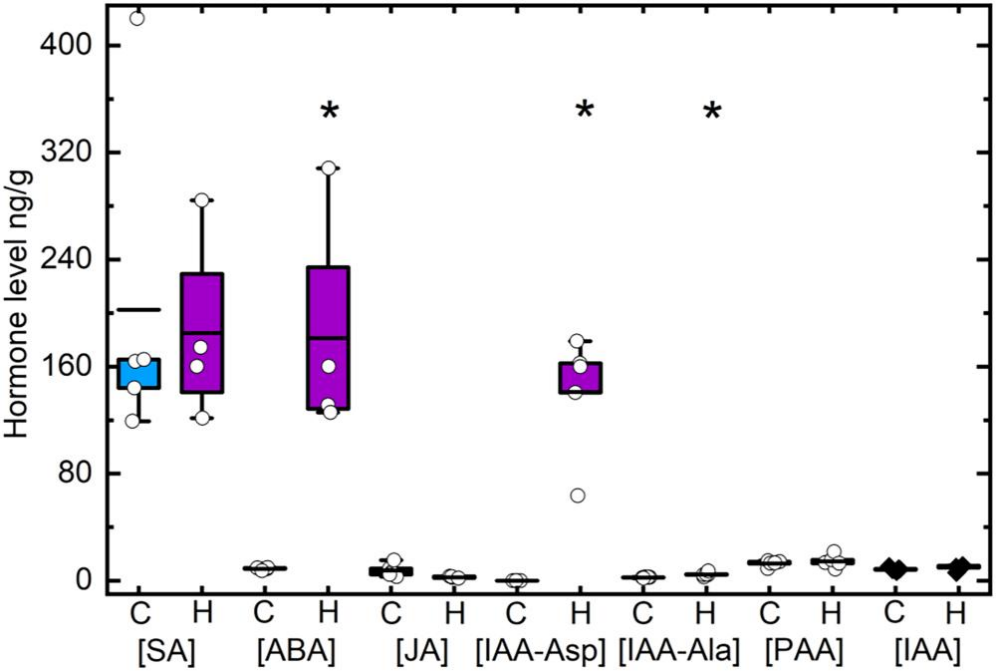
Measurements of hormone levels using LC-MS. SA, salicylic acid; ABA, abscisic acid; JA, jasmonic acid; IAA, indole-3-acetic acid (i.e. auxin); IAA-Asp, auxin-aspartate amino conjugate; IAA-Ala, auxin-alanine amino conjugate; PAA, phenylacetic acid. Asterisks indicate a significant difference at *P* < 0.05. Number of samples measured for each hormone and for each treatment is 4 or 5. C is control and H is heat stress treatment. Data points are overlayed in the box plots with the straight line representing the mean with the lower and upper edge representing the interquartile range (25% to 75%). Student's *t* tests (2-tailed, assuming equal variance) were used for *P* value calculation (*n* = 5).

The visible phenotype of dwarfing/stunting of all plant parts in *Setaria* grown under high temperature is striking (Fig. 1). To examine this phenotype in more detail, leaf hormone levels were profiled in heat-stressed and control plants (Fig. 9). The levels of salicylic acid (SA), jasmonic acid (JA), and indole-3-acetic acid (IAA) did not change significantly, nor did the level of phenylacetic acid (PAA), an auxin analog in plants (Fig. 9). However, the level of abscisic acid (ABA) increased markedly to about 20-fold over the control levels in the heat-stressed plant. Although the level of IAA did not change, there was massive accumulation of IAA-Aspartate amino conjugate in the heat-stressed plants while IAA-Aspartate amino conjugate is not detectable in the control plants. This compound is proposed to be an inactive reservoir for IAA (Ruiz Rosquete et al. 2012) or in one study a potent signal during abiotic stress (Ostrowski et al. 2016). In addition, there was also a significant accumulation of IAA-Alanine amino conjugate in the heat-stressed plants.

Transcriptional changes in genes involved in each of the hormone pathways, including SA, JA, ethylene (ET), auxin, ABA, cytokinin (CK), gibberellic acid (GA), and brassinosteroids (BRs) are presented in Supplementary Figs. S9 to S16. Overall, the transcriptional changes in genes involved in ABA and auxin pathways are in agreement with the trends observed for ABA and IAA levels as measured by LC-MS, suggesting an increase in ABA and a decrease in auxin signaling. Additionally, the transcriptome data support an upregulation of JA and BR signaling pathways, whereas a decrease in signaling through the SA, ET, auxin, GA, and CK is suggested by the transcript changes. Interestingly, the more pronounced changes in the SA and JA transcript pathways are not reflected as significant changes in the hormone levels as measured by LC-MS. Hormone analysis by LC-MS may suffer drawbacks of lower sensitivity and stability of the hormones, and the results here demonstrate the value of a systemic approach employing multiple platforms, which may provide complementary information.

## Discussion

### Effects of heat stress on photosynthesis and growth

The lack of effect on the rate of photosynthesis in response to CO_2_ and irradiance and on levels of photosynthetic proteins in the leaves of plants grown for 2 wk at 42/32 °C was unexpected (Figs. 2 and 3). The marked dwarfing observed (Fig. 1) suggests that carbon supply might be severely limiting. The thermal response of photosynthesis shown in Supplementary Fig. S2 suggests that C_4_ photosynthesis in *S. viridis* was relatively insensitive to the long-term exposure to elevated temperatures. Comparably, sorghum grown under long-term high temperatures (40 °C) showed no significant decrease in photosynthesis but decreases in chlorophyll a fluorescence and content were observed (Prasad et al. 2008). In another study, maize plants grown at 38 °C showed similar rates of photosynthesis but significant decreases in biomass (Perdomo et al. 2015). In contrast, sorghum grown at 44 °C displayed minor decreases in photosynthetic carbon assimilation (Prasad et al. 2006). Additionally, maize plants exposed to long-term heat stress of 35 °C had significant reduction photosynthesis and stomatal conductance (Killi et al. 2017). Likewise, maize plants grown at 400 ppm CO_2_ and 37 °C and at 370 ppm CO_2_ at ambient temperatures of 35 and 38.5 °C showed significant reductions in photosynthesis, respectively (Kim et al. 2007; Liu et al. 2022). Other examples of changes to photosynthesis in short-term exposure to heat stress include the decline in CO_2_ assimilation rates at temperatures above 38 °C in *Z. mays* (Crafts-Brandner and Salvucci 2002). In sorghum, photosynthesis declined after 3-h incubation at 40 °C (Yan et al. 2011). Similarly for *S. viridis* plants exposed to 40 °C for 4 h, reductions in CO_2_ assimilation were observed (Anderson et al. 2021). Several C_4_ species (monocot *Panicum coloratum* and *Cenchrus ciliaris*, and dicot *F. bidentis*) grown at moderately high temperatures (35/30 °C) also exhibited reduced photosynthetic capacity compared to plants grown at control temperatures (25/20 °C) (Dwyer et al. 2007). Other crop species such as cotton and wheat also exhibited significant reduction of photosynthesis at temperatures beyond 40 °C (Law and Crafts-Brandner 1999). However, in the present study, both *S. viridis* plants grown at control (28/22 °C) or high temperatures for 14 days (42/32 °C) suffered relatively little loss of photosynthetic activity at 45 °C (Supplementary Fig. S2).

Major points of high-temperature sensitivity have been identified in the photosynthesis pathway, including chloroplast electron transport components affected under high temperature and high light (Nash et al. 1985; Takahashi et al. 2004; Sharkey 2005; Allakhverdiev et al. 2008), and inhibition caused by RCA protein turnover, denaturation, and loss of activity (Sharkey et al. 2001; Salvucci and Crafts-Brandner 2004a, 2004b). However, despite transcriptome analyses identifying levels of many photosynthetic transcripts were reduced at high temperatures, this was only reflected in the proteomics profile in a small number of cases (Figs. 3 to 5). Lack of correlation between transcript levels and protein levels or nonlinear relationships between the 2 are not uncommon in the plant literature (Hajduch et al. 2010; Ponnala et al. 2014; Vélez-Bermúdez and Schmidt 2014). Some possible explanations for this lack of correlation are discussed below. First, mature, fully expanded leaf blades were used for this study. Depending on the stability of individual photosynthetic proteins (particularly under heat stress) and their developmental timing of synthesis, peak transcriptional activity may occur very early in leaf development and not coincide with protein level in mature tissue (Ponnala et al. 2014). Second, control of protein levels of some photosynthetic proteins encoded in the chloroplast, in particular those forming complexes in the chloroplast thylakoid, are believed to be controlled posttranscriptionally (Choquet and Wollman 2002; Eberhard et al. 2002; Park et al. 2012; Floris et al. 2013; Fankhauser and Aubry 2017; Zoschke and Bock 2018; Perdomo et al. 2021) and are often extremely long-lived proteins (Adam 1996; Bienvenut et al. 2011; Nelson and Millar 2015).

While there are examples of photosynthesis gene transcripts that correlate poorly or not at all with protein abundance, it is of interest to examine how broadly this occurs. Here, we have provided an additional in-depth analysis of Pearson correlation coefficients (*r*) and *r*^2^ values between log_2_(fold change) values of transcripts and proteins across 37 hierarchical functional categories and demonstrate that the magnitude and sign of correlations vary widely between functional categories. The strongest associations (all positive in sign) were observed for Calvin cycle (*r*^2^ = 0.87), protein degradation (ubiquitin-mediated) (*r*^2^ = 0.85), secondary metabolism (*r*^2^ = 0.73), major carbohydrate metabolism (*r*^2^ = 0.7), and redox categories (*r*^2^ = 0.7). In contrast, almost no transcript–protein association was observed among components of PSs I or II, TCA cycle, ribosomal proteins of 40S or 60S subunits of the 80S cytosolic ribosome, or lipid metabolism (all *r*^2^ < 0.1) pointing to posttranscriptional processes as the primary drivers of the response in those categories. That evolution would favor posttranscriptional mechanisms of control over these categories is consistent with their role in major energy fluxes and chemical energy/nutrient reserves that must respond rapidly to environmental fluctuations (Liu et al. 2016). A major influence of autophagy on lipid metabolism proteins has been reported in *Z. mays* (McLoughlin et al. 2018). Intriguingly, chloroplast ribosomal proteins showed a substantial negative correlation between transcript and protein response driven by decreases in proteins with elevated mRNA transcripts (*r* = −0.47, *r*^2^ = 0.22; Supplementary File S1). The Transcripto-Proteomic Correlation Analyzer (TPCA) Python tool used to perform and visualize these analyses (plus pregenerated interactive HTML scatter plot outputs) is available for download at https://github.com/phenospectral/TPCA.

The lack of obvious physiological effects on photosynthesis after 42 °C growth in these experiments suggests that the photosynthetic machinery of *S. viridis* had become thermally acclimated to 42 °C. Evidence for heat adaptation at 42 °C is supported by an increased *T*_opt_ for photosynthesis (Table 1), maintenance of photosynthesis rates at the high growth temperature and higher photosynthesis rates at 45 °C in the acclimated plants (Supplementary Fig. S2), maintenance of photosynthesis rates at the new *T*_opt_, and maintenance of photosynthetic capacity measured at a basal temperature of 25 °C (Fig. 2; Supplementary Fig. S2). These changes fulfilled all the criteria outlined by Way and Yamori (2014) of thermal acclimation of photosynthesis and suggest that a positive adjustment has occurred to optimize the plant response to heat stress. In this regard, there appears to have been some adaptive responses for a subset of photosynthetic proteins and a response to heat treatment in photosynthetic metabolite profiles (Figs. 3 and 4).

Notably, expression of chloroplast membrane transporters involved in C_4_ photosynthesis was suppressed at high temperatures at the transcript level and in the case of DCT2/DIT2 also at the protein level (Fig. 3). This may have been the cause for an increase in foliar malate levels at high temperature as DCT2/DIT2 is believed to move malate into the BS chloroplast (Weber and von Caemmerer 2010). However, photosynthetic malate probably only makes up a relatively small proportion of total leaf malate pools (Hatch 1979; Meister et al. 1996). In concert with a rise in malate levels, aspartate levels fell and levels of aspartate and alanine aminotransferase enzymes increased under heat (Fig. 3). This could be indicative of a shift toward aspartate formation from OAA in the mesophyll and its utilization in the BS to regenerate malate for decarboxylation as seen in the NADP-ME type C_4_ maize and *Flaveria* (Meister et al. 1996; Furbank 2011; Arrivault et al. 2017).

A shift toward aspartate as the translocated C_4_ metabolite under heat would likely be triggered by the strong induction of pathways implicated in combating oxidative stress (Supplementary Fig. S7). The reduction of oxidized glutathione and dehydroascorbate during detoxification of H_2_O_2_ occurs exclusively in the mesophyll compartment (Doulis et al. 1997), which would result in strong competition for reducing power in the mesophyll chloroplasts and reduced capacity for OAA reduction (Turkan et al. 2018). Furthermore, it can be hypothesized that there is a buildup of foliar nitrate under heat stress that would correspond to the observed increase in leaf nitrogen and downregulation of the gene expression in the nitrate assimilation pathway and contribute to the lack of reducing power in the chloroplast (Supplementary Fig. S8). Nitrate and nitrite reductase are localized to the mesophyll cells in NADP-ME-type C_4_ grasses (Moore and Black Jr 1979) consistent with competition for reducing power between OAA reduction, oxidative stress protection, and nitrogen metabolism in this compartment during heat treatment.

While adaptive responses in the C_3_ photosynthetic machinery and photorespiratory pathways of *Setaria* under high temperature were not generally large, the response of RCA expression (Fig. 3) was marked. RCA has been identified as a key point of heat sensitivity in the photosynthetic machinery of both C_3_ and C_4_ plants (Law and Crafts-Brandner 1999; Crafts-Brandner and Salvucci 2002; Salvucci and Crafts-Brandner 2004a, 2004b; Busch and Sage 2017; Scafaro et al. 2019; Degen et al. 2021). It has been proposed that the C-terminal extension is responsible for heat stability of RCA and the induction of the longer RCA isoform in heat-stressed *Setaria* supports this hypothesis (see Fig. 3 and Supplementary Fig. S3; Anderson et al. 2021). It has been shown that the RCA promoter for the *Setaria* alpha isoform has abiotic stress elements to induce expression at elevated temperatures (Kim et al. 2021); however, a deeper biochemical study is required as the short beta isoform of RCA has also been shown in numerous species to harbor thermotolerant characteristics (Kurek et al. 2007; Shivhare and Mueller-Cajar 2017; Degen et al. 2020). It is currently unknown whether the induction of the alpha isoform results in molecular stability of the RCA complexes at elevated temperatures and further investigation is required.

### Carbon partitioning and export

One of the largest metabolic effects of high-temperature growth of *Setaria* was on foliar carbohydrate levels (Figs. 6 and 7). A large shift occurred away from starch and in favor of high soluble sugar levels in leaves harvested midway through the photoperiod. Starch levels declined by almost two-thirds under heat treatment, sucrose more than doubled, and glucose and fructose levels increased more than 10-fold (Fig. 6). This was in part due to changes in levels of key enzymes in carbohydrate synthesis but presumably also due to reduced sucrose export, as SWEET13 expression was severely reduced under heat stress (Fig. 8). While compartmentation of these carbohydrates between M and BSC was not determined, nor was subcellular location, these are massive changes in carbon allocation with likely far reaching impact on osmotic regulation and sugar signaling pathways (Li and Sheen 2016; Saddhe et al. 2021). Examination of the transcript and proteome profiles performed here shed some light on the mechanisms underlying these changes in carbohydrate levels.

The control on the rate of sucrose synthesis is thought to be mainly exerted through regulations on the activities of cFBPase and SPS in both C_3_ and C_4_ plants (Lunn and Furbank 1999). Here the levels of cFBA, cPGI, cPGM, UGP2, and SPP proteins, which are all part of the sucrose synthesis pathway, significantly increased (Fig. 8), which is consistent with an increase in sucrose biosynthesis. However, there was no change in the amount of cFBPase and SPS proteins and their transcript abundances significantly decreased (Fig. 8). This may indicate the importance of posttranslational regulation of cFBPase and SPS activity.

The increased accumulation of sucrose in the leaves of heat-stressed plants could additionally be caused by reduced phloem loading. Phloem loading of sucrose is thought to occur by export of sucrose into the apoplasm from the BS by SWEET13 and subsequent loading into the phloem by SUT1 (Slewinski et al. 2009; Bezrutczyk et al. 2018, 2021). While these proteins are difficult to detect in the proteomic profiles, transcript levels were significantly decreased in the heat-stressed plants (Fig. 8), suggesting a reduced capacity for phloem loading in heat stress. Impairments in sucrose transport in C_4_ plants have been previously linked to a dwarf phenotype (Russin et al. 1996; Ma et al. 2009; Slewinski et al. 2009; Bezrutczyk et al. 2018), although, unlike our experiments, feedback inhibition of photosynthesis resulting from sugar accumulation in leaves and evidence of foliar oxidative stress were obvious in these cases.

The shift toward hexose accumulation in heat-treated leaves was presumably the result of increased sucrose breakdown either by SUS or invertase. Under heat treatment, vacuolar invertase expression increased substantially at both the transcript and protein level and SUS also increased (Fig. 8). This would suggest a shift toward vacuolar storage of hexoses in heat-treated plants. Reduced starch accumulation did not appear to be due to reduced expression of genes encoding enzymes in starch biosynthesis (Fig. 8) but likely due to allosteric regulation of the AGPase (Cross et al. 2004; Hwang et al. 2005). Activity of this enzyme is tightly controlled by the relative levels of 3-PGA (an activator of the enzyme) and Pi (an inhibitor). Under heat treatment, Pi levels increased and 3-PGA levels declined significantly (see Fig. 3), which would reduce activity of this key regulatory enzyme.

The significance of the buildup of soluble sugars seen here in response to heat could in fact be in a protective and acclimation response to heat stress. Accumulation of solutes such as sugars and proline seen here is thought to provide osmoprotection of cellular processes under stress through their role as compatible solutes retaining turgor and potentially stabilizing proteins (Garg et al. 2002; Lunn et al. 2014; Siddique et al. 2018). In addition to the shift from starch to sucrose and hexose, levels of raffinose rose markedly under heat treatment (Fig. 7). Raffinose oligosaccharides are regarded as important molecules in stress response in plants, because of their membrane-stabilizing, antioxidant, and possible predicted signaling functions (Hincha et al. 2003; Panikulangara et al. 2004; Kim et al. 2008; Nishizawa et al. 2008; Valluru and Van den Ende 2011). The increases in raffinose oligosaccharides in our experiments are consistent with highly significant increases in expression of transcripts encoding raffinose synthase, stachyose synthase, and galactinol synthase (Fig. 7; Supplementary Fig. S6) to stimulate accumulation of raffinose to combat heat damage.

### Effects of hormone and sugar signaling on growth and development

**Figure 10.**
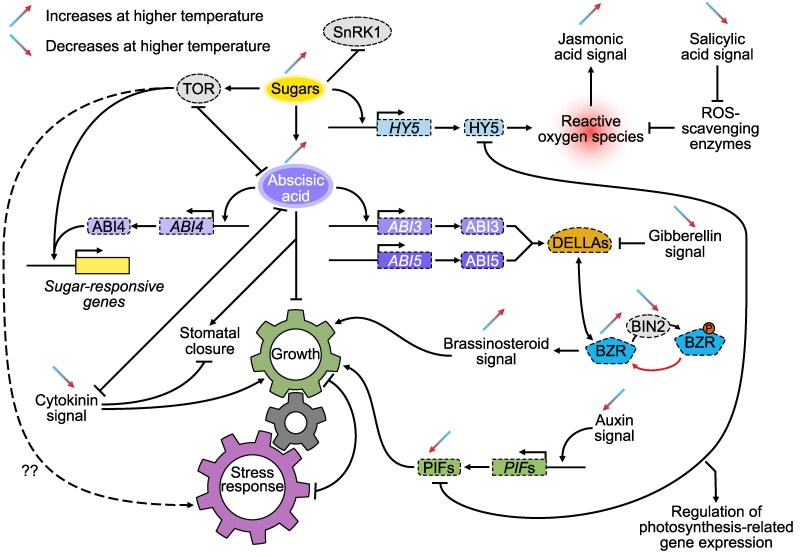
A hypothetical model of the regulation of growth and heat stress response by phytohormones and sugar signaling in *S. viridis*. Arrows indicate a positive relationship where one component may stimulate/stabilize the other component. Lines with a blunt end indicate an inhibitory relationship, where one component negatively interacts/regulates the other component. ABA, abscisic acid; CK, cytokinin; GA, gibberellin; BR, brassinosteroid; SA, salicylic acid; JA, jasmonic acid; ROS, reactive oxygen species; TOR, target of rapamycin; SnRK1, sucrose nonfermenting related kinase 1; HY5, long hypocotyl 5; BZR, brassinosteroid positive regulator related; BIN2, brassinosteroid insensitive 2; ABI4, ABA insensitive 4; PIF, phytochrome interacting factor.

Taken together, these changes in carbon partitioning indicate that *Setaria* is responding strongly to the high-temperature conditions and adapting its metabolism very effectively. However, while photosynthesis appears to be protected under heat treatment, growth is certainly not. The stunting of these plants under high temperature without signs of leaf stress or photosynthetic inhibition is suggestive of a very effective homeostatic response in plant growth and minimization of vegetative damage to the plant from heat stress. Long-term heat stress applied to both maize and sorghum plants have also been reported to result in reductions in plant height (Obata et al. 2015; Smith et al. 2023). A hypothesis is presented below (Fig. 10) which attempts to link metabolite levels and gene expression to sugar and hormone signaling in mediating a coordinated response to heat stress, which restricts growth and minimizes damage.

Increases in ABA levels commonly observed under abiotic stress are thought to inhibit growth by stabilizing DELLA proteins, which repress the growth promotive effects of GA (Szostkiewicz et al. 2010; Blanco-Touriñán et al. 2020) and act broadly across the ABA-dependent pathway of stress response (Chen et al. 2020). This ABA-dependent pathway appeared to dominate the signaling response in the experiments described here, as evidenced by a 20-fold increase in ABA levels, upregulation of gene expression of enzymes in ABA biosynthesis, and increased expression of proteins known to be controlled by ABA levels (Supplementary Fig. S9). Among the pathways regulated by ABA is the TOR signaling pathway (Wang et al. 2018). It has been reported that ABA accumulated following stress binds to intracellular PYR/PYL/RCAR receptors and ABA-receptor complexes bind to and inhibit PP2C protein phosphatases (Wang et al. 2018). This releases the activity of Snf1-related protein kinase 2s (SnRK2s). The SnRK2s phosphorylate downstream targets to elicit protective responses such as stomatal closure and the expression of ABA responsive genes (Chen et al. 2020). The elevated SnRK2 activity is thought to inhibit the growth promoting action of TOR to sacrifice growth for survival during stress (Fu et al. 2020). In the current data set, stomatal conductance was not substantively reduced after heat treatment (Supplementary Fig. S2), which may reflect acclimation following a short-term effect of ABA. Note that expression of 2 of the 3 OST1 (OST1/SnRK2) genes in *Setaria* thought to be involved in ABA-induced stomatal control during stress in other species (Acharya et al. 2013) decreased under heat treatment while one was increased (Supplementary Fig. S9).

In addition to engagement of a strong ABA-dependent growth response in *Setaria* under heat treatment, concerted action of reactive oxygen produced under high-temperature stress is also known to trigger increases in JA and affect a range of growth-related targets. Increases in JA can directly affect the action of the DELLA proteins via protein stability and posttranslational modification (Liu and Timko 2021). This effect of JA would provide additional repression of growth through the GA pathway if both ABA and JA were acting to stabilize DELLA protein levels. While increases in JA were not apparent in this study, measurements of JA are challenging and strong evidence for a JA response is provided by the upregulation of gene expression for a range of JA-induced transcripts (Supplementary Fig. S10).

Along with the strong response of ABA targets in the transcriptional profiles after heat treatment (Supplementary Fig. S9), the transcriptional response of auxin target transcripts suggested a fall in auxin levels (Supplementary Fig. S11). While free IAA did not change after heat stress, there was a marked increase in amino-conjugates of auxin (Fig. 9). A buildup of these conjugates has also been seen in concert with increased ABA levels in heat-stressed wheat anthers (Bheemanahalli et al. 2020) and salt-stressed pea seedlings (Ostrowski et al. 2016). Amino-conjugates are traditionally regarded as an inactive “reservoir” and homeostatic mechanism for regulating free IAA (Bartel 1997), but it is also thought that these conjugates may be potent signaling compounds themselves under stress (Rosquete et al. 2013), also reducing growth and development. For example, IAA conjugate treatments strongly inhibit IAA-induced shoot growth and root initiation in tomato cell cultures (Magnus et al. 1992). There is little information in the literature on how these conjugates may function in stress signaling.

While there is strong evidence from the data shown here for a concerted downregulation of growth via hormone signaling, the large changes in carbon partitioning away from starch and toward soluble sugars (Figs. 6 and 8; Supplementary Fig. S4) are more challenging to interpret in a signaling context. The large increases in hexose levels seen here following heat stress would be expected to feedback on photosynthetic gene expression and trigger phenotypes similar to those seen when leaf sugar export is impeded or exogenous sugars are fed to leaves (Azcón-Bieto 1983; Goldschmidt and Huber 1992; Van Huylenbroeck and Debergh 1996; Van Oosten et al. 1997; Pego et al. 2000). However, while there was some global transcriptional downregulation of photosynthetic transcripts under heat, this was often not reflected in protein levels or physiological phenotypes (Figs. 2 to 5). There is little published evidence for sugar feedback on photosynthetic gene expression in C_4_ leaves (Lunn and Furbank 1999; Lobo et al. 2015; Marquardt et al. 2021) with results implying that C_4_ plants are more sensitive to sugar starvation than feedback inhibition (Henry et al. 2020).

Large increases in soluble sugar levels would also be expected to have a profound effect on TOR signaling acting to promote TOR activation of ribosome biogenesis, cell division, and growth (Li and Sheen 2016; Haq et al. 2022; Jindal et al. 2022). Indeed, a highly significant (*P* < 2.2E^−16^) positive correlation (*r* = 0.44) was observed between the previously published responses of Arabidopsis TOR-regulated transcripts to a 2-h treatment with 15 mm glucose in *Arabidopsis thaliana* seedlings (Xiong et al. 2013) and the responses of their *S. viridis* orthologs to prolonged heat stress in this study (Supplementary Fig. 17A). Conversely, a highly significant (*P* < 2.2E^−16^) *negative* association (*r* = −0.41) was observed when the analogous comparison (Supplementary Fig. 17B) was made with a published response of *A. thaliana* seedlings to treatment with the powerful TOR inhibitor, AZD8055 (Dong et al. 2015). This was not evident in the response to high sugar levels in *S. viridis* grown at high irradiance (Henry et al. 2020) or inhibition of TOR action in *S. viridis* by chemical means (Da Silva et al. 2021), suggesting that TOR sensing in C_4_ plants may be less sensitive to sugar regulation than in C_3_. The negative feedback regulation of TOR signaling was also recently reported (Jindal et al. 2022), which may also explain why increased sugar levels are not always associated with TOR activation and stimulation of plant growth. It should also be noted that the majority of studies on TOR and regulation of growth and carbohydrate status have been done in sink tissue, not photosynthetic source tissue. The role of TOR in these tissues may differ substantially from that in tissues receiving externally supplied photosynthate.

### Major declines in ribosomal protein levels point to potential impairment of ribosomal biogenesis

Activation of TOR is typically associated with transcriptional upregulation of ribosome biogenesis when TOR signaling is experimentally modulated in plants (Mahfouz et al. 2006; Ren et al. 2011; Chen et al. 2018; Soprano et al. 2018; Busche et al. 2021). We observed strong coordinated transcriptional upregulation of ribosome biogenesis with the Gene Ontology (GO) terms “translation” and “ribonucleoprotein complex biogenesis” being highly significantly overrepresented among upregulated transcripts under heat stress (Supplementary File S1, Supplementary Data Set 2, and Tables S3 to S5). In contrast, at the protein level, ribosomal proteins tended to be decreased in abundance with 83 (69%) of the 121 quantified “ribosomal protein”-annotated proteins being decreased in abundance by between 20% and 90% and only 6 (5%) being increased in abundance by more than 20% (Supplementary Data File 1 and Table S2). Prolonged (37 °C) heat stress has recently been shown to cause impairment of ribosome biogenesis in Arabidopsis by compromising the processing of ribosomal RNA (rRNA) precursors in the nucleolus (Darriere et al. 2022). A similar impairment of ribosome biogenesis in *S. viridis* may contribute to the major decline in ribosomal protein levels observed here. Such an impairment would be expected to be detrimental to growth by decreasing the energy efficiency of ribosome biogenesis and growth and may contribute to the impaired growth phenotype observed in *S. viridis* under prolonged heat stress in this study.

Almost all of the observed increases in ribosomal protein abundance were marginal (up to 1.6-fold). Interestingly, however, the abundance of one particular ribosomal protein, RPL10A (encoded by Si026830m), increased by over 20-fold (Supplementary File S1, Data Set 2 and Table S2). It has recently been shown that RPL10A expression is upregulated by ABA and that RPL10A may be a positive effector of ABA-mediated responses in Arabidopsis (Ramos et al. 2020). It would be interesting to explore the role of ABA-mediated RPL10A accumulation in the *S. viridis* heat stress response via mutant studies. It remains unclear from our results whether decreased ribosome abundance is a negative consequence of thermal disruption to ribosome biogenesis/stability or a survival-promoting response orchestrated by complex homeostatic sensing and signaling networks involving TOR and ABA. Either way, it is clear that the reduced plant growth observed under heat stress in this study may be explained at least in part by reduced protein synthesis capacity.

## Conclusion

Long-term high-temperature stress of *S. viridis* induces a strong “dwarf” phenotype, which is likely a result of a converging set of hormonal signals all impacting to reduce growth. Remarkably, photosynthetic processes were quite robust under high temperature and appear to have been protected from damage by acclimation of carbohydrate metabolism, oxidative stress responses, and potentially by flexibility in the production of aspartate versus malate in the C_4_ photosynthetic pathway in this species under conditions where the mesophyll chloroplast stroma was experiencing oxidizing conditions.

## Materials and methods

### Plant growth and anatomical measurements

Seeds of *Setaria viridis* genotype A10 were treated in 10% liquid smoke for 2 h at room temperature to promote germination. Seeds were sown onto a rice mix (prepared by CSIRO) in a small clear plastic container (10 cm × 10 cm × 5 cm) with the lid closed to prevent drying. Seeds were germinated in a Conviron growth chamber under 16 h/8 h day–night cycle, and the temperature was set at 28 °C day/20 °C night, relative humidity 40% to 60%, CO_2_ at 380 ppm, and an irradiance at 1,000 *μ*mol quanta m^−2^ s^−1^. After germination, seedlings were transplanted into individual pots made with PVC plastic pipe that are 10 cm in diameter and 75 cm tall containing the same rice mix. At 2 wk old, 20 plants were transferred into a Conviron growth chamber that had the exact same condition except the temperature was set at 42 °C day/32 °C night. Plants were watered daily in the morning and fertilized with diluted Seasol liquid fertilizer 3 times a week.

At the end of the treatment, 3 plants from the control and heat-stressed group were taken for destructive analysis. The roots were washed and the roots and shoots cut separately with scissors and placed in paper bags. The material was dried in an 80 °C oven for 3 d and the weight of the dried material measured. One fully expanded fresh leaf from each plant was taken for fixation for anatomical analysis. The leaf was equally divided into tip, middle, and bottom sections and placed in 25% glyceraldehyde solution in glass vials. Leaf sections were finely sliced by hand and visualized using a microscope attached to a camera.

### Gas exchange measurements

Gas exchange was measured on the flag leaf of the 4-wk-old control and heat-stressed plants using a LI-6400XT with a LED light source (Licor). Measurements were performed early in the morning, from ∼2 h into the day cycle. Measured leaves were initially equilibrated under standard condition of 380 *μ*mol mol^−1^ CO_2_, 25 °C leaf temperature, 2,000 *μ*mol quanta m^−2^ s^−1^, and a flow rate of 500 *μ*mol s^−1^, until leaf photosynthesis stabilized. Photosynthetic CO_2_ response (*A*/C_i_) curves were performed by first stepwise decrease in CO_2_ partial pressure from 400 to 0 *μ*mol mol^−1^ and then stepwise increase from 0 to 1,500 *μ*mol mol^−1^ while maintaining leaf temperature at 25 °C and irradiance of 2,000 *μ*mol m^−2^ s^−1^. Photosynthetic light response curves were performed by stepwise increasing the light intensity from 0 *μ*mol quanta m^−2^ s^−1^ to 2,000 *μ*mol m^−2^ s^−1^ while maintaining CO_2_ partial pressure at 380 *μ*mol mol^−1^ and leaf temperature at 25 °C. Leaf dark respiration measurements were performed with dark adapted plants that just exited the night cycle (Supplementary Data Set 3). Leaves were first measured at 25 °C, 380 *μ*mol mol^−1^ CO_2_. Then leaves were measured at 20, 25, 30, 35, 40, and 45 °C. Measurements were taken at each temperature when leaf dark respiration stabilized in the new condition. At the end of the gas exchange experiments, leaf disks were taken from the measured leaves (4 plants for each condition) and snap-frozen in liquid nitrogen for measurement of enzyme activity, chlorophyll content, starch content, and protein content. Two-tailed Student’s *t* test (2-sample equal variance) was used for statistical analysis (*n* = 4).

### Sampling of *S. viridis* leaf disks for subsequent biochemistry and omics analyses

To ensure minimal impact of diurnal variation in sampling time, leaf disks (typically 0.5 cm^2^) were taken in the middle of the light cycle. For all omic sampling requirements, fully expanded leaves were chosen for harvesting and pooled prior to specific extraction methods.

### Enzyme activity assays

Rubisco and PEPC assays were performed as previously published (Sharwood et al. 2016). Leaf disks (0.5 cm^2^) were punched from the midsection of fully expanded leaves from the control and heat-stressed plants were collected and snap-frozen in liquid nitrogen. One leaf disk was homogenized with a glass homogenizer in 500 *μ*L of freshly prepared ice-cold extraction buffer (100 mm HEPES-KOH, pH 7.4, 5 mm DTT, 0.1% BSA, 0.05% Triton X-100, 2 mm EDTA, 5 mm MgCl_2_, 1% PVPP) plus 10 *μ*L of protease inhibitor cocktail (P-9599; Sigma, St. Louis, MO, USA). The homogenate was quickly transferred into a 1.5-mL tube and centrifuged at top speed for 30 s at 4 °C. To cuvettes 1 and 2, 435 *μ*L of freshly prepared Rubisco assay buffer (100 mm EPPS, pH 8, 20 mm MgCl_2_, 1 mm EDTA, 10 mm NADH, 10 mm ATP, 50 mm coupling enzyme, 400 mm NaHCO_3_) was added; to cuvette 3, 435 *μ*L of freshly prepared PEPC assay buffer (100 mm EPPS, pH 8, 20 mm MgCl_2_, 1 mm EDTA, 40 mm NADH, 5 mm glucose-6-phosphate, 400 mm NaHCO_3_, 0.5 unit of malate dehydrogenase enzyme) was added. Enzyme activity was determined by the consumption of NADH at 340 nm using a diode array spectrophotometer at 25 °C (Agilent). Two-tailed Student's *t* test (2-sample equal variance) was used for statistical analysis (*n* = 4).

### Leaf elemental analysis and chlorophyll and carbohydrate content

Leaf chlorophyll content of the leaves was measured following the method developed by Porra et al. (1989). Leaf tissue was homogenized using a glass homogenizer in ice-cold 80% acetone buffered with 2.5 mm phosphate buffer (pH 7.8). The homogenate was centrifuged at 12,000 × *g* for 10 min at 4 °C, and 100 *μ*L of the supernatant was diluted with 900 *μ*L of the extraction buffer before reading the absorbance at 750, 663.6, and 646.6 nm on a spectrophotometer.

Leaf disks from the heat stress experiment were oven-dried and wrapped into a small aluminum weighing boat with their accurate weight recorded. The percentage of carbon and nitrogen relative to leaf dry weight was estimated by combustion of samples in an elemental analyzer (EA1110, Carlo Erba).

Leaf starch content was determined for a leaf disk (with known area) that was collected from the midsection of a fully expanded mature leaf from the heat stress experiment. The fresh weight of the leaf disk was determined before extraction. The Megazyme Total Starch Assay Kit was used for starch determination following the manufacturer’s instructions. Two-tailed Student's *t* test (assuming equal variance) was used for statistical analysis (*n* = 4).

### RNA extraction and quality analysis

Total RNA was extracted from frozen leaf powder (from pooled midsection of fully expanded leaves, 5 control plants, 6 heat-stressed plants) using TRIzol Reagent. For each tube of homogenized leaf sample (∼100 mg), 1 mL of TRIzol Reagent was mixed in and incubated at room temperature for 5 min to lyse the cells. The cell lysate was centrifuged at 12,000 × *g* for 15 min at 4 °C to remove debris, and the supernatant was transferred to a new tube. To each tube, 0.2 mL of chloroform was added, and the tubes were shaken by hand for 15 s and incubated for 2 to 3 min at room temperature. The mixture was centrifuged at 12,000 × *g* for 15 min at 4 °C, and the upper aqueous phase containing the RNA was transferred into a new tube. For RNA precipitation, 0.5 mL of isopropanol was added to the aqueous phase and incubated at room temperature for 10 min. The mixture was centrifuged at 12,000 × *g* for 10 min at 4 °C, and the supernatant was discarded. The resulting RNA pellet was washed with 75% ethanol and centrifuged at 7,500 × *g* for 5 min at 4 °C. The wash was repeated 3 times. The RNA pellet was resuspended in 50 *μ*L of RNAse-free water.

The quality and accurate concentration of the extracted RNA was measured by Agilent 2100 Bioanalyzer using the Agilent RNA 6000 Nano Kit following the manufacture's instruction.

### RNA sequencing and library preparation

Isolated RNA sample was sent to the Next Generation Sequencing Facility at the Hawkesbury Institute for the Environment at the Western Sydney University for RNA-seq library construction and sequencing. Ribosomal RNA was removed before the construction of strand-specific RNA library using Illumina TruSeq RNA Sample Preparation kit version 2. Data were obtained for 5 control samples and 6 heat-stressed samples.

### RNA sequencing data analysis

The quality of the RNA-seq data was checked using the FASTQC program (https://www.bioinformatics.babraham.ac.uk/projects/fastqc/). Reads were quality trimmed, and adapter sequences were removed using Trimmomatic (Bolger et al. 2014) with the following setting: LEADING:10 TRAILING:10 SLIDINGWINDOW:4:15 MINLEN:50 (and the rest of the settings are default). This setting means the first and the last 10 bases are cut off if their quality score is below the threshold; the average score of a sliding window size of 4 bases is calculated and the read trimmed if the average score falls below 15; read is discarded if read length is below 50. The quality trimmed reads were mapped to the genome of *S. italica* (v2.1) (downloaded from http://plants.ensembl.org/) using TopHat2 with the default setting allowing 2 mismatches (Kim et al. 2013). The alignments were counted to exons using HT-Seq with mode set to union (Anders et al. 2015). The read count data were analyzed in DESEQ for differential expression analysis (Anders and Huber 2010). Differential expression analysis was performed and fold changes (heat stress over control) was calculated and log_2_ transformed. Multiple testing correction was performed by the Benjamini–Hochberg procedure and FDR set to 5%. Annotation file for *S. italica* genome was downloaded from Phytozome (https://phytozome.jgi.doe.gov/pz/portal.html#). The file contains the orthologous gene information for each *Setaria* gene, and orthologous gene from Arabidopsis, rice, and maize was assigned to each *Setaria* gene. GO terms were assigned to each gene using the rice GO annotation, and the genes were further annotated with MapMan mappings (http://mapman.gabipd.org/mapmanstore). The raw data and metadata are uploaded to GEO, accession number GSE216993. Processed data are presented in Supplementary Table S1.

### Proteomics analysis

Total leaf protein was extracted (5 control plants, 5 heat-stressed plants) using the tricarboxylic acid (TCA)/acetone method (Damerval et al. 1986). Homogenized leaf tissue (∼300 mg from each sample) from the heat stress experiment (same sample used for RNA extraction) was extracted with 1 mL of cold precipitation buffer (10% w/v trichloroacetic acid, 0.07% v/v 2-mercaptoethanol in acetone, prechilled at −20 °C overnight). The mixture was incubated at −20 °C for at least 2 h (or overnight). The homogenate was centrifuged at 19,000 × *g* for 15 min at 4 °C, and the supernatant was discarded using a pipette. The pellet was washed 3 times with ice-cold acetone (with 0.07% 2-mercaptoethanol) to remove chlorophyll, and the pellet was dried under nitrogen gas. The pellet containing protein was resuspended in solubilization buffer (8 m urea, 2% w/v CHAPS, 30 mm Tris-HCl pH 7.8, use approximately 60 *μ*L per milligram of protein) with the help of a sonicator water bath. The mixture was centrifuged at 14,000 × *g* to remove insoluble debris, and the supernatant containing protein was collected. The extracted protein solution was quantified using the 2-D Quant Kit (GE Healthcare) following the manufacturer's instruction.

Disulfide bonds were reduced by adding solubilization buffer to 2 mg of protein to a total volume of 995 *μ*L. Fresh DTT (1 m) was made by dissolving a small amount of powder in 50 mm ammonium bicarbonate (NH_4_CO_3_), and 5 *μ*L of 1 m DTT was added to each tube of protein solution. The solution was incubated at 37 °C for 1 h for complete reduction. During the incubation, iodoacetamide to 40 mm was added (Pieterse et al. 2001) and incubated in the dark for 2 h at room temperature. An additional 5 *μ*L of 1 m DTT was added to the tubes to stop the alkylation, and the tubes were incubated at room temperature for 15 min. For protein digestion, the proteinase enzyme Lys-C (Pierce Lys-C, MS grade) was added at a 1:100 enzyme:protein (w/w) ratio and incubated at 37 °C for 4 h. The samples were diluted 1:8 with 50 mm NH_4_CO_3_ to reduce the urea concentration to 1 m. CaCl_2_ was also added to each sample to a final concentration of 1 mm. The proteinase enzyme Trypsin (Pierce Trypsin, MS grade) was added to the tubes at an enzyme:protein ratio (w/w) of 1:30 and incubated in a 37 °C water bath overnight. The samples were cooled on ice, and the digestion was stopped by adding trifluoroacetic acid to 1% v/v. The tubes were centrifuged at 2,500 × *g* for 10 min at room temperature, and the supernatant was collected. The volume was reduced by drying the samples in a Labconco CentriVap SpeedVac System and stored at −20 °C for further processing.

Dried peptide samples were reconstituted in 1 mL of 5% formic acid. The reconstituted peptides were labeled following the on-column stable isotope dimethyl labeling protocol (Boersema et al. 2009).

The labeling reagent was prepared by combining 4.5 mL of sodium phosphate buffer pH 7.5 (prepared by mixing 1 mL of 50 mm NaH_2_PO_4_ with 3.5 mL of 50 mm Na_2_HPO_4_) with 250 *μ*L of 4% (v/v) formaldehyde in water (CH_2_O or CD_2_O) and 250 *μ*L of 0.6 m sodium cyanoborohydride in water (NaBH_2_CN or NaBD_3_CN). For the heat stress experiment, the light label was introduced to the peptide samples from the control plants, and the intermediate label was introduced to the peptide samples from the heat-stressed plants. Sep-Pak C18 cartridges (Waters) were engaged onto a vacuum extraction manifold (Waters). Each cartridge was washed with 2 mL of acetonitrile and conditioned with 2 mL of reverse-phase solvent A (0.6% [v/v] acetic acid). Peptide samples (500 *μ*g equiv.) were loaded onto each cartridge, and the cartridge was washed with 2 mL of reverse-phase solvent A. The labeling reagent was added to the cartridges, 1 mL at a time for a total of 5 mL for each sample and was allowed to flow through the cartridges slowly. The cartridges were again washed with 2 mL of reverse-phase solvent A, and the labeled peptide sample was eluted with 500 *μ*L of reverse-phase solvent B (0.6% [v/v] acetic acid and 80% [v/v] acetonitrile) and collected into a Lo-bind protein tube.

### MS

The peptide samples were dried in a SpeedVac and submitted to the Sydney Mass Spectrometry facility (The Charles Perkins Centre, The University of Sydney) for untargeted MS analysis. Reconstituted peptide (1 *μ*L) was injected into an in-house built fritless nano 75 *μ*m × 30 cm column packed with ReproSil Pur 120 C18 stationary phase (1.9 *μ*m, Dr. Maisch GmbH, Germany) fitted on a Thermo Scientific Ultimate 3000 HPLC and autosampler system for separation, before introduced into a Q Exactive Plus mass spectrometer (Thermo Fisher Scientific, Waltham, MA, USA). Peptide fractions were directed for MS through a nanoelectrospray interface, and charged species were captured and the Orbitrap mass analyzer was used to acquire full MS with *m*/*z* range of 350 to 1,550. The 20 highest peaks were fragmented by applying C-trap dissociation collision and tandem MS were generated. The MS acquisition time was 60 min in total for each sample.

### Proteomics data analysis

The generated “.raw” files containing acquired data were analyzed using the freely available proteomics software MaxQuant (version 1.5.2.8) (Cox and Mann 2008; Tyanova et al. 2016a). The *S. italica* protein sequence v2.1 (Bennetzen et al. 2012) (downloaded from http://plants.ensembl.org/) was used for library searching of the peptides. Peptide modifications selected include DiMethy Lys0/DiMethy N-term0 (for light-labeled peptides) and DiMethy Lys4/DiMethy N-term4 (for intermediate-labeled peptides), and the level of multiplicity is 2, to achieve quantitation. The mass error tolerance for the main peptide search was 10 ppm. Other search parameters are kept default as recommended in Tyanova et al. (2016a). About 1,800 protein groups with quantitative information (the ratio between intermediate- and light-labeled peptides) were obtained and further analyzed in the Perseus software (Tyanova et al. 2016b). Mainly, the protein ratio of log_2_ transformed, statistical testing was performed using single sample *t* test to test if the log_2_-transformed signal intensity ratios were significantly different from 0 in Microsoft Excel. Additionally, when a protein group was only detected in less than 3 out of the 5 samples submitted, the ratio was determined as insignificant and not used. Processed data are presented in Supplementary Table S1 and the complete data set submitted to MassIVE database (MassIVE Dataset ID: MSV000096617; ProteomeXchange Dataset ID: PXD058688; doi:10.25345/C5W08WV0K).

### Metabolite analysis

About 100 mg of frozen leaf powder samples (the same material used for RNA and protein analysis) for extraction was weighed into a prefrozen 2-mL Eppendorf tube containing a steel ball bearing without thawing. An accurate weight was recorded for each sample for use later in data normalization. The tissues were further homogenized in a TissueLyser II bead mill (Qiagen; Cat. No. 85300) for 1 min at 20 Hz without thawing out. Then, each tube containing frozen tissue powder was added 5 volumes (5 *μ*L per mg fresh weight of tissue) of cold (−20 °C) Metabolite Extraction Medium (85% [v/v] HPLC grade MeOH [Sigma], 15% [v/v] untreated MilliQ H_2_O, 8.7 *µ*g mL^−1^ ribitol as internal standard), and tubes were vortexed briefly, rapidly transferred to an Eppendorf Thermomixer Comfort (Eppendorf; Cat. No. 5355 000.011) and heated to 65 °C and shaken at 1,400 rpm for 15 min. Tubes were then centrifuged at 20,000 × *g* for 10 min to pellet insoluble material, and 80% of the supernatant was transferred into a new clear 2-mL round-bottom polypropylene Eppendorf tube and stored as a stock extract at −80 °C until further analysis.

From each metabolite extract, 15 *μ*L was aliquoted into a glass vial insert and dried in a centrifugal vacuum concentrator (Labconco CentriVap, Catalog Number 7811030). Dried metabolite extracts were chemically derivatized by methoximation and trimethylsilylation on a Gerstel MPS2XL Multipurpose Sampler (Gerstel) operating in the PrepAhead mode for automated online derivatization and injection. The derivatization procedure consisted of the following steps: (i) addition of 10 *μ*L of 20 mg mL^−1^ methoxyamine hydrochloride (Supelco, Catalog Number 33045-U) in anhydrous derivatization-grade pyridine (Sigma-Aldrich, Catalog Number 270970) and incubation at 37 °C for 90 min with agitation at 750 rpm; (ii) addition of 15 *μ*L of derivatization-grade *N*-methyl-*N*-(trimethylsilyl)trifluoroacetamide (MSTFA; Sigma-Aldrich; Catalog Number 394866) and incubation at 37 °C for 30 min with agitation at 750 rpm; and (iii) addition of 5 *μ*L of alkane mix (0.006% [v/v] *n*-dodecane, 0.006% [v/v] *n*-pentadecane, 0.006% [w/v] *n*-nonadecane, 0.006% [w/v] *n*-docosane, 0.006% [w/v] *n*-octacosane, 0.006% [w/v] *n*-dotriacontane, and 0.006% [w/v] *n*-hexatriacontane] dissolved in anhydrous pyridine and incubation for 1 min at 37 °C with agitation at 750 rpm. Samples were injected into the GC/MS instrument immediately after derivatization.

Derivatized metabolite samples were analyzed on an Agilent 5975C GC/MSD system comprised of an Agilent GC 7890N gas chromatograph (Agilent Technologies, Palo Alto, CA, USA) and 5975C Inert MSD quadrupole MS detector (Agilent Technologies, Palo Alto, CA, USA). The GC was fitted with a 0.25 mm ID, 0.25 *μ*m film thickness, and 30 m Varian FactorFour VF-5 ms capillary column with a 10 m integrated guard column (Varian, Inc., Palo Alto, CA, USA; Product No. CP9013). Samples were injected into the split/splitless injector operating in splitless mode with an injection volume of 1 *μ*L, an initial septum purge flow of 3 mL min^−1^ increasing to 20 mL min^−1^ after 1 min, and a constant inlet temperature of 230 °C. Helium carrier gas flow rate was held constant at 1 mL min^−1^. The GC column oven was held at the initial temperature of 70 °C for 1 min before being increased to 325 °C at 15 °C min^−1^ before being held at 325 °C for 3 min. Total run time was 21 min. Transfer line temperature was 250 °C. MS source temperature was 250 °C. Quadrupole temperature was 150 °C. Electron impact ionization energy was 70 eV, and the MS detector was operated in full scan mode in the range of 40 to 600 *m*/*z* with a scan rate of 3.6 Hz. The MSD was pretuned against perfluorotributylamine (PFTBA) mass calibrant using the “atune.u” autotune method provided with Agilent GC/MSD Productivity ChemStation Software (Revision E.02.01.1177; Agilent Technologies, Palo Alto, CA, USA; Product No. G1701EA).

### GC/MS data processing

All GC/MS data were processed using the online MetabolomeExpress data processing pipeline4 (Carroll et al. 2010). Raw GC/MS files were exported to NetCDF format using Agilent MSD ChemStation software (Revision E.02.01.1177; Agilent Technologies, Palo Alto, CA, USA; Product No. G1701EA). Peak detection settings were as follows: Slope threshold = 200; Min Peak Area = 1000; Min. Peak Height = 500; Min. Peak Purity Factor = 2; Min. Peak Width (Scans) = 5; Extract Peaks = on. Peaks were identified by MSRI library matching that used retention index and mass spectral similarity as identification criteria. MSRI library matching parameters were as follows: RI Window = ± 2 RI Units; MST Centroid Distance = ± 1 RI Unit; Min. Peak Area (for peak import): 5000; MS Qualifier Ion Ratio Error Tolerance = 30%; Min. Number of Correct Ratio Qualifier Ions = 2; Max. Average MS Ratio Error = 70%; Remove qualifier ion not time-correlated with quantifier ion = OFF; Primary MSRI Library = “Carroll_2014_Arabidopsis_Photorespiration_Mutants.MSRI”; Add Unidentified Peaks to Custom MSRI Library = OFF; Use RI calibration file specified in metadata file = ON; Carry out per-sample fine RI calibration using internal RI standards = OFF.

The Carroll_2014_Arabidopsis_Photorespiration_Mutants.MSRI primary library contains entries derived manually from analyses of authentic metabolite standards run under the same GC/MS conditions as the biological samples as well as entries for unidentified peaks that were automatically generated by MetabolomeExpress while processing the data from the reference photorespiration mutants. Library matching results were then used to construct a metabolite × sample data matrix with peak areas being normalized to internal standard (i.e. ribitol). As a quality control filter, samples were checked for the presence of a strong ribitol peak with a peak area of at least 1 × 10^5^ and a deviation from the median internal standard peak area (for that GC/MS batch sequence) of less than 70% of the median value. Statistical normalization to tissue mass was not required because chemical normalization to tissue mass had already been carried out by adjusting extraction solvent volume proportionally to tissue mass. The heat versus control signal intensity ratio of each metabolite was calculated by dividing the mean (normalized) signal intensity of each metabolite in the heat-stressed plants by its mean (normalized) signal intensity in the control plants. Statistical significances were calculated by 2-tailed Welch's *t* tests (*n* = 5) in the MetabolomeExpress Comparative Statistics tool. The full data set has been uploaded to the MetaboLights metabolomics data repository (Haug et al. 2020) under the accession number MTBLS8842. Processed data are presented in Supplementary Data Set 1.

### Hormone analysis using UPLC-Orbitrap

Samples from 5 biological replicates for the control and 5 biological replicates for the heat-stressed plants were analyzed. The frozen tissue samples (∼100 mg weighed into a tube with fresh weight recorded) were homogenized in a TissueLyser II bead mill (Qiagen; Cat. No. 85300) for 1 min at 20 Hz without thawing out. For auxin extraction (Ng et al. 2015), 20 *μ*L of the internal standard (1 *μ*g/mL of 3-[2H5]indolylacetic acid) followed by 1 mL extraction solvent (20% methanol:79% propanol:1% glacial acetic acid) was added to each tube, and the tubes were incubated in a sonicator bath for 15 min at 4 °C. Samples were then centrifuged at 16,100 × *g* for 15 min. The supernatant was transferred to a fresh tube and dried in a centrifugal vacuum concentrator (Labconco Centrivap). Extraction was repeated and the supernatant combined with the first batch and dried again. Dried samples were resuspended in methanol/water (60:40, v/v) and filtered with a Nanosep MF GHP (hydrophilic polypropylene) 0.45-*μ*m filter (Pall Life Sciences) prior to injection. For extraction of ABA, JA, and SA (Miyazaki et al. 2014; Xu et al. 2016), 20 *μ*L of the internal standards ([^2^H_6_]ABA or 1 *μ*g mL^–1^ dihydrojasmonic acid and 2-hydroxybenzoic acid) followed by 950 *μ*L of extraction solvent (70:30, acetone: 50 mm citric acid) was added to each tube of frozen leaf powder. Tubes were placed on a shaker at 4 °C in the dark for 5 h, and then the tubes were left uncapped in a fume hood to allow the acetone layer to evaporate overnight. The ABA, JA, and SA in the remaining aqueous phase were extracted by partitioning 3 times with 500 *μ*L of diethyl ether. The ether phase was collected in a 2-mL glass vial (Kinesis Australia Pty, Redland Bay, Qld) and evaporated using a centrifugal vacuum concentrator (Labconco Centrivap) until dry. Dried samples were resuspended in 60% methanol (50 *μ*L) and filtered with a Nanosep MF GHP (hydrophilic polypropylene) 0.45-*μ*m filter (Pall Life Sciences) prior to injection. Samples and standards (5 *μ*L) were injected onto an Agilent Zorbax Eclipse 1.8 *μ*m XDB-C18 2.1 × 50 mm column. For ABA, SA, and JA, the column temperature was held constant at 45 ± 0.5 °C. Solvent A consisted of 0.1% aqueous formic acid, and solvent B consisted of methanol with 0.1% formic acid. JA, SA, and ABA were eluted with a linear gradient from 10% to 50% solvent B over 8 min and 50% to 70% solvent B from 8 to 12 min (then held at 70% from 12 to 20 min) at a flow rate of 200 *μ*L min^−1^. For auxins, the column temperature was 35 ± 0.5 °C and the linear gradient was the same as above using solvent A consisting of 0.1% aqueous formic acid and solvent B of 90% methanol/water with 0.1% formic acid. Solvents were LC-MS grade from Fisher Chemical. The eluted phytohormones from the column were introduced into the mass spectrometer via a heated electrospray ionization (HESI-II) probe and analyzed using the Q Exactive Plus Orbitrap (Thermo Scientific, Waltham, MA, USA). The HESI was operated in the negative ion polarity for JA, SA, and ABA with parameters as follows: the electrospray voltage was 2.5 kV, and the ion transfer tube temperature was 250 °C. The vaporized temperature and the S-lens RF level were 300 °C and 50 V, respectively. Ultrahigh purity nitrogen was used as the sheath gas, auxiliary gas, and sweep gas at flows of 45, 10, and 2 L min^−1^, respectively. For auxins, the HESI was operated in the positive ion polarity and the electrospray voltage was set to 3.5 kV. Positive or negative ion polarity tandem MS was carried out using targeted parallel reaction monitoring with a mass resolution of 17,500 at 1.0 microscan. The Automatic Gain Control target value was set at 1.0E^+05^ counts, maximum accumulation time was 50 ms, and the isolation window was set at *m*/*z* 4.0. Data were acquired and analyzed using the Thermo Scientific Xcalibur 4.0 software.

### GO enrichment analysis

The list of gene IDs for significantly up- and downregulated genes (padj < 0.05) in the heat-stressed plants were submitted to the agriGO website (Du et al. 2010) (http://systemsbiology.cau.edu.cn/agriGOv2/) for functional characterization using its singular enrichment analysis function. The *Setaria italica* v2.1 database was used to assign GO terms to genes. Only the list of expressed genes (mean RPKM > 20 in both of the conditions) was used as the reference background when considering enrichment. A GO category was considered significantly overrepresented when the hypergeometric test *P* < 0.05, and a Benjamini–Yekutieli FDR < 0.1, and at least 5 entries mapped. To reduce the complexity of the GO data set, the list of significant GO terms in the biological process category was input into REViGO (Supek et al. 2011) to remove functional and semantic redundancies. Hierarchical clustering was further performed with the refined list of GO in REViGO using the default setting.

### Global analysis of hormone-regulated genes

The transcriptional responses of hormone-regulated genes in the heat-stressed *Setaria* plants were compared to the hormone-associated responses in Arabidopsis. The Arabidopsis hormone treatment data sets were kindly curated and provided by Dr. Michael Groszmann, which he obtained from the AtGenExpress database (atpbsmd.yokohama-cu.ac.jp/AtGenExpressJPN/AtGenExpress.html). These data consist of microarray-based gene expression responses to exogenous treatment by various hormones in Arabidopsis seedlings. The method of analysis was adopted from Groszmann et al. (2015) with variations to address species differences. To perform the comparison between *Setaria* and Arabidopsis, the list of expressed (RPKM >20) *Setaria* gene IDs was converted to Arabidopsis gene IDs by identifying the closest related ortholog. This was done using the *S. italica* genome annotation file version 2.1 from Phytozome (https://phytozome.jgi.doe.gov/pz/portal.html). The list of orthologous Arabidopsis gene IDs was mapped to the list of expressed genes from the Arabidopsis hormone treatment microarray data set to identify a common list of genes expressed in both experiments (Arabidopsis hormone and *Setaria* heat), forming the “working set.” For analysis of each hormone response, the list of upregulated (stimulated) and downregulated (repressed) genes in response to a particular hormone treatment was analyzed separately. Count data were obtained for hormone-stimulated genes that were also upregulated in the heat experiment and for hormone-stimulated genes that were downregulated experiment, under the condition that the genes exist in the “working set,” so that only genes expressed in both experimental conditions were compared. Similarly, count data were obtained for hormone-repressed genes by comparing to upregulated and downregulated gene lists from the heat experiment. To avoid the situation where multiple *Setaria* genes can be mapped to the same Arabidopsis ID, thereby inflating the count due to gene duplication, redundancy in Arabidopsis ID in the up- and downregulated gene list from the heat experiment was removed, so that each Arabidopsis gene was only compared once in each scenario. The count data formed the basis of contingency tables for carrying out Fisher's exact test to identify overrepresentation of hormone-regulated genes in the *Setaria* heat stress treatment (*P* < 0.01 was considered significant).

### Accession numbers

Sequence data from this article can be found in the GEO (Gene Expression Omnibus) database under accession number GSE216993.

## Supplementary Material

koaf005_Supplementary_Data

## Data Availability

The data underpinning this article will be shared on reasonable request to the corresponding author. Processed data is available in supplementary materials. Transcriptome data is publicly available on GEO under the accession: GSE216993. Metabolite data is publicly available in the MetaboLights repository under the accession: MTBLS8842. The complete proteome dataset is publicly available in MassIVE under the id: MSV000096617.

## Supplementary data

The following materials are available in the online version of this article.

**Supplementary Figure S1.** The anatomical characteristics of fully expanded leaves of the plants grown under control and heat conditions by light microscopy.

**Supplementary Figure S2.** Thermal response of photosynthesis parameters.

**Supplementary Figure S3.** Amino acid sequence alignment of the 2 RCA genes differentially expressed under the 2 growth conditions.

**Supplementary Figure S4.** Representative transmission electron microscopy images showing the accumulation of starch granules in mature leaves of the “Control” “Control to Heat,” and “Heat” plants.

**Supplementary Figure S5.** Summary of all the identified metabolites detected by GC/MS showing changes in plants subjected to 2 wk of heat stress.

**Supplementary Figure S6.** Changes in the raffinose pathway induced by heat treatment.

**Supplementary Figure S7.** Response of genes involved in oxidative stress to long-term heat stress in *S. viridis*.

**Supplementary Figure S8.** Changes associated with genes encoding for proteins involved in nitrogen assimilation and metabolism under heat stress.

**Supplementary Figure S9.** ABA transcriptional response pathways.

**Supplementary Figure S10.** JA transcriptional responses.

**Supplementary Figure S11.** Analysis of gene expression in auxin biosynthesis and degradation.

**Supplementary Figure S12.** Transcriptional changes associated with SA response pathway under heat stress in *S. viridis*.

**Supplementary Figure S13.** The ethylene-related transcriptional responses.

**Supplementary Figure S14.** Transcriptional response associated with CKs.

**Supplementary Figure S15.** The GA transcriptional response under heat.

**Supplementary Figure S16.** The transcriptional response associated with BRs in the heat-stressed *S. viridis*.

**Supplementary Figure S17.** Cross-species correlation analysis between transcriptional responses of *A. thaliana* TOR-regulated genes and those of their *S. viridis* orthologs.

**Supplementary Table S1.** The complete RNA-seq data set for the heat stress experiment.

**Supplementary Table S2.** Protein groups identified by LC-MS.

**Supplementary Table S3.** Complete GO list for significantly upregulated genes in the heat-stressed plants produced by agriGO.

**Supplementary Table S4.** Complete GO list for significantly downregulated genes in the heat-stressed plants produced by agriGO.

**Supplementary Table S5.** The most significantly enriched GO terms in the upregulated genes after removing functional and semantic redundancies in REViGO.

**Supplementary Data Set 1.** Responses of leaf metabolites to 2 wk of heat stress in *S. viridis* as measured by GC/MS metabolomics.

**Supplementary Data Set 2.** Systematic analysis of correlations between the responses of *S. viridis* leaf transcripts and proteins in major functional categories to 2 wk of heat stress.

**Supplementary Data Set 3.** Dark respiration measurements and modeling of thermal responses.

**Supplementary File S1.** HTML files associated with Supplementary Data Set 2.

## Dive Curated Terms

The following phenotypic, genotypic, and functional terms are of significance to the work described in this paper:

RCA Gramene: AT2G39730
RCA Araport: AT2G39730

DIT1 Gramene: AT5G12860
DIT1 Araport: AT5G12860

APL1 Gramene: AT5G19220
APL1 Araport: AT5G19220

APS1 Gramene: AT3G22890
APS1 Araport: AT3G22890

PRK Gramene: AT1G32060
PRK Araport: AT1G32060

PETC Gramene: AT4G03280
PETC Araport: AT4G03280

PPDK Gramene: Sobic.009G132900
PPDK Araport: Sobic.009G132900

ATPD Gramene: AT4G09650
ATPD Araport: AT4G09650

PEPC Gramene: Sobic.010G160700
PEPC Araport: Sobic.010G160700

TPT Gramene: At5g46110
TPT Araport: At5g46110

NADH CHEBI: CHEBI:16908

PEPCK Gramene: Sobic.001G432800
PEPCK Araport: Sobic.001G432800

UDP Gramene: Uridine diphosphate
UDP Araport: Uridine diphosphate

nitrate CHEBI: CHEBI:17632
