# Supplementary material for: Systems analysis of long-term heat stress responses in the C4 grass Setaria viridis
Source: Plant Cell. 2025 Jan 8;37(4):koaf005. doi: 10.1093/plcell/koaf005 (PMC11964294; doi:10.1093/plcell/koaf005)
Supplement: koaf005_Supplementary_Data [file koaf005_supplementary_data.zip › Supplementary Figures S1S17 and Methods.pdf]

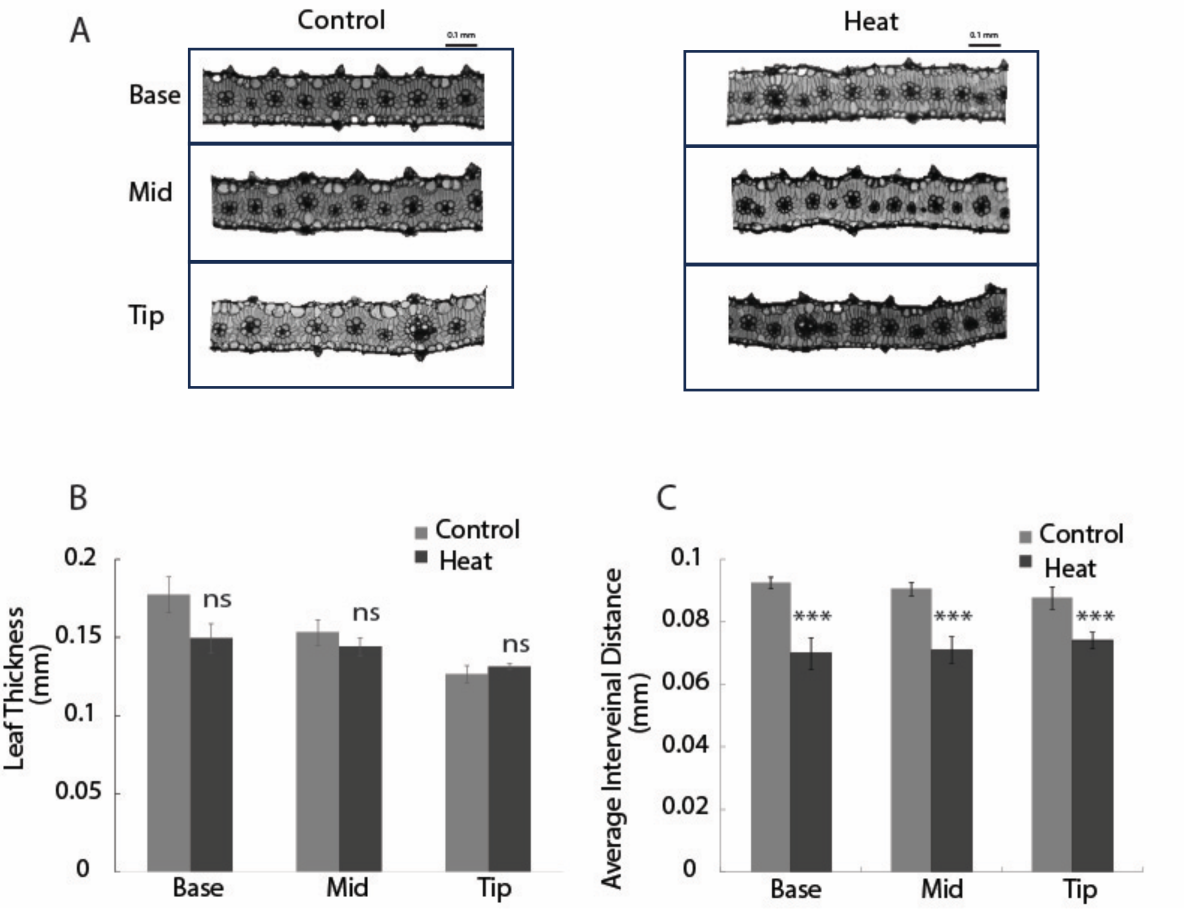

Supplementary Figure S1: The anatomical characteristics of fully expanded leaves of the plants grown under control and heat conditions by light microscopy. A) Representative pictures of the base, mid, and tip sections of fully expanded leaves. Images extracted to greyscale for a white background. Scale bar applies to all images. B) The measured interveinal distance at the base, mid and tip of the leaves. Number of plants used for each treatment was 3, and 3 hand-cut sections of the base, mid and tip were made from each leaf, 5 measurements of interveinal distance were made in each image to obtain the average. Error bars shown represent SEMs, and p-values were calculated using Student's t-tests, where "ns" indicates not statistically significant and "***" indicates statistical significant with $p < 0.05$.

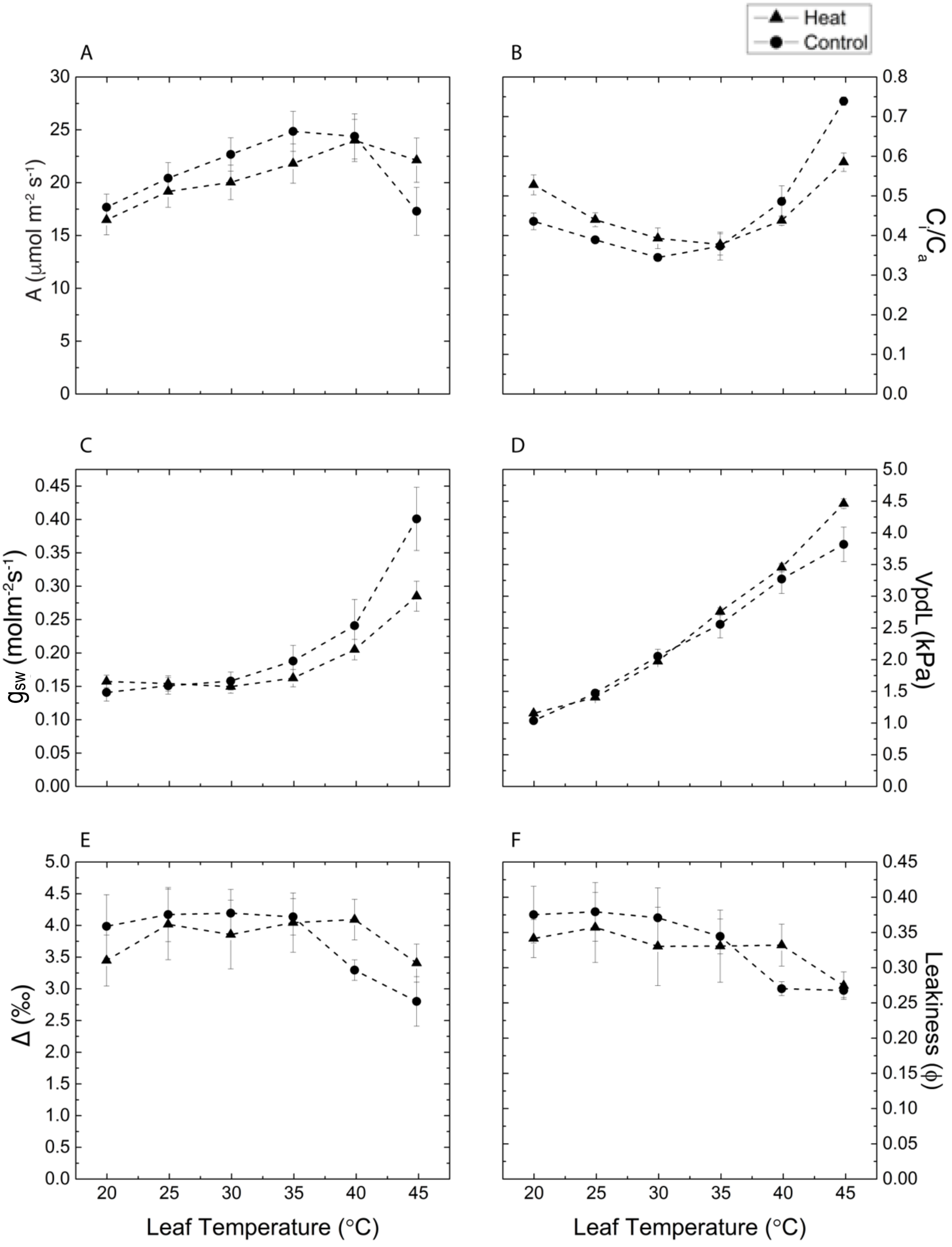

Supplementary Figure S2: Thermal response of photosynthesis parameters. Temperature response of CO_2 assimilation rate (A), C_i/C_a (B), stomatal conductance (C), leaf vapor pressure deficit (D), carbon isotope discrimination Δ (E), and Bundle Sheath Leakiness φ (F) measured at ambient CO_2 concentration (380 μmol.mol^{-1}), 2000 μmol.m^{-2}s^{-1} light intensity and various temperatures. Error bars represent SEMs. The on-line carbon isotope discrimination of the fully expanded leaves of the control and heat-stressed plants were measured using LI-6400XT systems coupled to a tunable diode laser (TDL; model TGA100, Campbell Scientific, Inc., Logan, UT, USA) as described in Pengelly et al. (2010). Measured leaves were allowed to reach a steady state photosynthesis inside the Licor chamber at an ambient CO_2 concentration of 380 μmol.mol^{-1}, an irradiance of 2000 μmol m^{-2}.s^{-1}, a flow rate of 500 μmol.s^{-1} and 25 ºC. The leaf temperature was then adjusted stepwise from 20 ºC, to 25 ºC, 30 ºC, 35 ºC, 40 ºC and 45 ºC. For the TDL, N_2 and O_2 were used as input air, and were mixed using

mass flow controllers (OMEGA Engineering). Part of the input air and air from a compressed air tank were used to correct for gain drift throughout the day. A calibration gas was made by mixing 10% CO_2 ($d^{13}C$ = -24.5‰) and part of the input air in a calibration gas mixing system to generate ~1000 μmol.mol^{-1} CO_2. From this, six different CO_2 concentrations with the same isotopic composition was generated by mixing with the input air. The CO_2 cylinders used in the Licor had a carbon isotope composition of 13.1 ‰ with respect to PDB. The TDL measurements followed a sequence of 20s measurements of zero and six calibration CO_2 concentrations, the compressed air, the reference and sample gases of the two Licor machines that were connected. The reference air from the Licor were collected from the tube used to match the infrared gas analysers (IRGAs) in the Licor. The gases from the Licor were dried by passing through a dryer assembly in the gas line before entering the TDL for $d^{13}C$ measurements.

Estimation of the Bundle Sheath CO_2 leakiness:

Carbon isotope discrimination (D) was calculated as (Evans et al., 1986):

$$\Delta = \frac{\xi\,(\delta o - \delta e)}{1+\delta o-\xi(\delta o - \delta e)} \qquad (1)$$

where, δ_e and δ_o are the carbon isotope compositions of dry air entering and leaving the leaf chamber, respectively, measured by the TDL. $\xi = C_e/(C_e - C_o)$, and C_e and C_o are the CO_2 partial pressures of dry air entering and leaving the chamber, respectively, measured by the TDL.

CO_2 leakiness (φ) was estimated using the model of C_4 carbon isotope discrimination developed by Farquhar (1983):

$$\Delta = \alpha_b \frac{(C_a - C_s)}{C_a} + \frac{C_s - C_i}{C_a} + (e_s + \alpha_1)\frac{C_i - C_m}{C_a} + (b_4' + (b_3' - s)\phi)\frac{C_m}{C_a} \qquad (2)$$

Where, C_a, C_s, C_i and C_m are the CO_2 partial pressures in the air, at the leaf surface, in the intercellular airspace, and in the mesophyll cytoplasm, respectively. a_b is the fractionation factor during diffusion through the boundary layer (2.9 ‰), a is the fractionation associated with diffusion of CO_2 in air (4.4 ‰), α_1 is the fractionation during dissolution of CO_2 (1.1 ‰), a_1 is the fractionation during aqueous diffusion (0.7 ‰), and s is the fractionation during CO_2 leakage from the bundle sheath cells (1.8 ‰).

The combined fractionation of Rubisco, respiration, and photorespiration, b'_3 is given by:

$$b_3' = b_3 - \frac{e(M_m + M_s)}{V_c} - \frac{fV_o}{V_c} \qquad (3)$$

where V_c and V_o are the rates of Rubisco carboxylation and oxygenation, respectively. M_m and M_s are the rates of respiration occurring in the mesophyll and bundle sheath cells, respectively. b_3 is the fractionation factor by Rubisco (30 ‰) and f is the fractionation associated with photorespiration (11.6 ‰). e is the fractionation associated with respiration (-5.1 ‰), and was calculated from the difference between the $d^{13}C$ in the CO_2 cylinder (-13.1 ‰) used during experiments and that in the atmosphere under growth conditions (-8 ‰) (Tazoe et al., 2009). The combined fractionation by PEP carboxylation, respiration, and fractionation during dissolution of CO_2 and conversion to HCO_3^- is given by:

$$b_4' = b_4 + \left(h - e_b - b_p\right)\frac{V_p}{V_h} - \frac{eM_m}{V_p} \qquad (4)$$

where, V_p and V_h are the rates of PEP carboxylation and CO2 hydration. $b_4 = b_p + e_b + e_s$ (-5.7 ‰ at 25°C) is the combined fractionation of PEP carboxylation (b_p = 2.2 ‰) and the preceding isotopic equilibrium fractionation (e_b = -9‰ at 25 °C) and dissolution of CO_2 (e_s), and h is the fractionation of the catalyzed CO_2 hydration (1.1 ‰) (Henderson et al., 1992; Cousins et al., 2006).

To calculate φ, it was assumed that both the boundary layer conductance and the internal conductance to CO_2 diffusion were large ($C_a = C_s$ and $C_i = C_m$) and that there was sufficient

carbonic anhydrase activity such that V_p/V_h = 0. And since measurements were made at 2% O_2, it was also assumed that V_o = 0. To account for the contribution of respiration in Equations (3) and (4), it was assumed that M_m + M_s = R_d, the rate of measured dark respiration, and that M_m = 0.5 R_d. Using the C_4 photosynthesis model (von Caemmerer, 2000), V_c and V_p were approximated by V_c = A + R_d and V_p = (A + 0.5 R_d)/(1 − ϕ), respectively, where A is the CO_2 assimilation rate. With these simplifications:

$$b_3' = b_3 - e\,\frac{R_d}{A-R_d} \quad (5)$$

and

$$b_4' = b_4 - e\,\frac{0.5\,R_d(1-\varphi)}{A+0.5R_d} \quad (6)$$

Using Equations (3) to (6), Equation (2) can be modified to form:

$$\Delta = \alpha + \frac{(e_s+\alpha_1-\alpha)A}{g_m{*}C_a} + \left[b_4 - e\,\frac{0.5R_d}{A+R_d} + \left(b_3 - s - e\left(\frac{R_d}{A+R_d} - \frac{0.5R_d}{A+0.5R_d}\right)\right)\phi - \alpha\right] * \frac{C_i - \frac{A}{g_m}}{C_a} \quad (7)$$

where g_m is the mesophyll conductance to CO_2 diffusion and $A/g_m = C_i - C_m$.
Rearranging Equation (7) gives an explicit expression of ϕ:

$$\phi = \frac{\left[\left(\Delta - \alpha - \frac{(e_s+\alpha_1-\alpha)A}{gmCa}\right) - \left(b_4 - e\frac{0.5R_d}{A=0.5R_d} - \alpha\right)\left(\frac{C_i - \frac{A}{gm}}{Ca}\right)\right]}{\left[b_4 - e\frac{0.5R_d}{A+R_d} + \left(b_3 - s - e\left(\frac{R_d}{A+R_d} - \frac{0.5R_d}{A+0.5R_d}\right)\right)\left(\frac{C_i - \frac{A}{gm}}{Ca}\right)\right]} \quad (8)$$

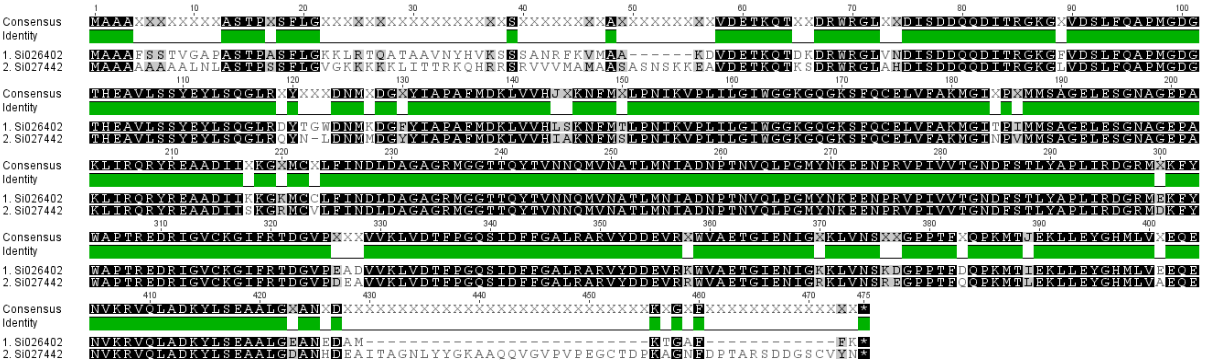

Supplementary Figure S3: Amino acid sequence alignment of the two Rubisco Activase genes differentially expressed under the two growth conditions. The major isoform Si026402m was more highly expressed under control condition (28 ºC day / 22 ºC night). The minor isoform Si027442 was very strongly upregulated under heat stress condition (42 ºC day / 32 ºC night).

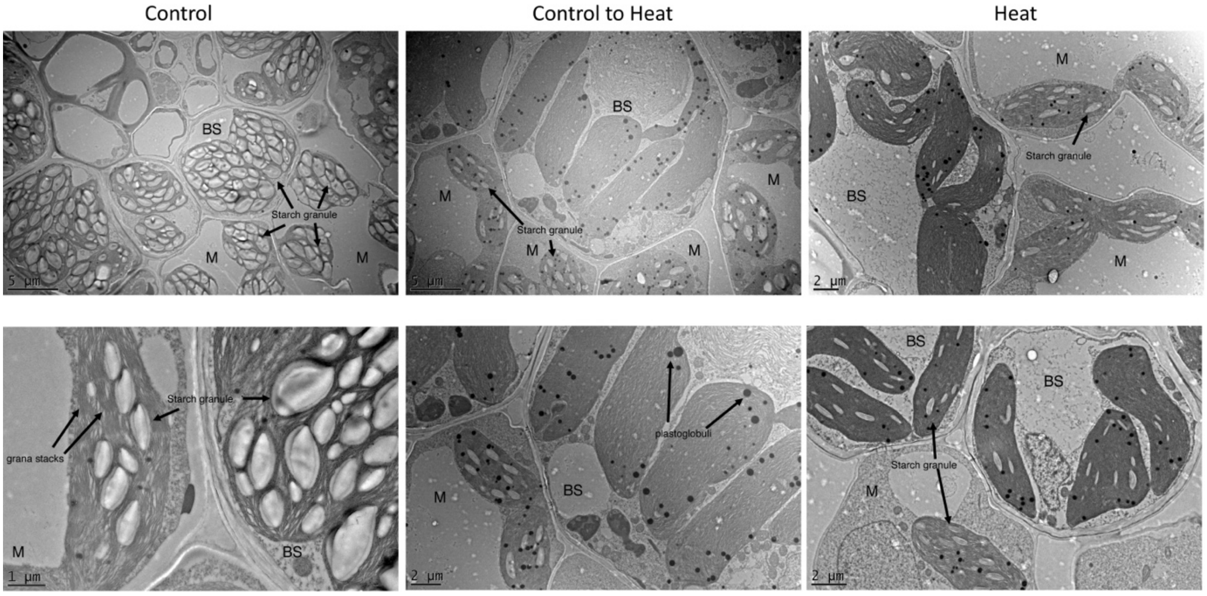

Supplementary Figure S4: Transmission electron microscopy images showing the accumulation of starch granules in mature leaves of the "Control", "Control to Heat", and "Heat" plants. The leaves from "Control" plants developed fully at 28°C day / 22°C night. The leaves from "Control to Heat" plants developed fully at 28°C day / 22°C but were transferred to 42°C day / 32°C night chamber for two weeks. The leaves from "Heat" plants developed fully at 42°C day / 32°C night. Top and bottom panels are images of the same region of different magnifications. Three plant samples were imaged for each condition.

Chemical Class	Name of compound	Compound retention time/identifier peak	Setaria viridis A10 (leaf) * 42degreeC_2 week / Setaria viridis A10 (leaf) * 28degreeC_2 week	p-val
Amino acid	L-Alanine	L-Alanine (3TMS)_ID33_RI1357_MZ188	1.75	1.71E-01
Amino acid	L-Asparagine	L-Asparagine (3TMS)_ID93_RI1670.5_MZ188	0.76	3.20E-01
Amino acid	L-Aspartic acid	L-Aspartic acid (3TMS)_ID58_RI1508.5_MZ218	0.15	1.02E-02
Amino acid	L-Isoleucine	L-Isoleucine (2TMS)_ID20_RI1287.4_MZ158	1.62	1.80E-02
Amino acid	L-Methionine	L-Methionine (2TMS)_ID59_RI1514.8_MZ176	0.82	4.64E-01
Amino acid	L-Phenylalanine	L-Phenylalanine (2TMS)_ID84_RI1631.9_MZ218	1.59	2.03E-01
Amino acid	L-Proline	L-Proline (2TMS)_ID22_RI1297_MZ142	2.28	1.30E-04
Amino acid	L-Threonine	L-Threonine (2TMS)_ID21_RI1292.7_MZ219	0.94	6.95E-01
Amino acid	L-Valine	L-Valine (2TMS)_ID14_RI1210.5_MZ144	1.14	3.64E-01
Amino acid	Ornithine	Ornithine (4TMS)_ID118_RI1812.2_MZ174	1.41	1.22E-01
Amino acid derivative	2-methylserine	2-methylserine (3TMS) _ID15_RI1219_MZ160	8.54	2.55E-02
Amino acid derivative	Beta-Alanine	beta-Alanine (3TMS)_ID49_RI1424_MZ248	0.38	3.00E-04
Amino acid derivative	Gamma-Aminobutyric acid	4-Aminobutyric acid (3TMS)_ID63_RI1524.8_MZ174	1.99	4.64E-01
Amino acid derivative	L-Alpha-aminobutyric acid	2-Aminobutyric acid (2TMS)_ID4_RI1165.5_MZ130	2.14	2.76E-02
Amino acid derivative	L-Homoserine	L-Homoserine (2TMS)_ID32_RI1356.4_MZ146	2.39	1.57E-01
Amino acid derivative	Pyroglutamic acid	Pyroglutamic acid (2TMS)_ID62_RI1522.1_MZ156	0.87	2.32E-01
Amino acid derivative	Shikimic acid	Shikimic acid (4TMS)_ID115_RI1799_MZ204	0.92	8.45E-01
Antioxidant	alpha-Tocopherol	alpha-Tocopherol (1TMS)_ID211_RI3151.3_MZ502	5.62	4.00E-04
Antioxidant	L(+)-Ascorbic acid	L(+)-Ascorbic acid (4TMS)_ID138_RI1936.8_MZ332	3.22	1.14E-01
Antioxidant derivative (ascobate break	Threonic acid	Threonic acid (4TMS)_ID64_RI1545.8_MZ292	0.84	4.84E-02
GABA	p-Aminobenzoic acid	p-Aminobenzoic acid (2TMS)_ID121_RI1839.4_MZ266	0.61	4.61E-02
Mineral	Phosphoric acid	Phosphoric acid (3TMS)_ID18_RI1263.9_MZ314	1.77	1.21E-03
Organic acid	Glucuronic acid	Glucuronic acid methoxime (5TMS) EZ Peak 1_ID135_RI1919.4_MZ333	1.56	1.32E-01
Organic acid	Malonic acid	Malonic acid (2TMS)_ID10_RI1197.9_MZ75	0.96	7.19E-01
Organic acid/TCA/C4	Malic acid	Malic acid (3TMS)_ID235_RI1475.5_MZ233	1.53	2.99E-02
Photorespiration	Glycolic acid-2-phosphate	Glycolic acid-2-phosphate (3TMS)_ID74_RI1588.5_MZ357	0.81	5.19E-01
Photorespiration/Amino acid	Glycine	Glycine (3TMS)_ID23_RI1302.1_MZ248	0.73	2.92E-01
Photorespiration/Amino acid	L-Glutamic acid	L-Glutamic acid (3TMS)_ID82_RI1615.3_MZ246	0.62	2.06E-02
Photorespiration/Amino acid	L-Glutamine	L-Glutamine (3TMS)_ID111_RI1773.4_MZ156	0.46	7.74E-02
Photorespiration/Amino acid	L-Serine	L-Serine (2TMS)_ID17_RI1254.4_MZ132	0.49	3.85E-03
Photorespiration/TCA/Calvin	3-Phosphoglyceric acid	Glyceric acid-3-phosphate (4TMS)_ID114_RI1796.4_MZ357	0.34	2.59E-03
Photorespiration/TCA/Calvin	Glyceric acid	Glyceric acid (3TMS)_ID27_RI1322.4_MZ292	2.1	4.21E-03
Polyamine	Putrescine	Putrescine (4TMS)_ID106_RI1739.2_MZ214	2.54	2.72E-03
Sugar	Arabinose	Arabinose methoxime (4TMS)_ID91_RI1667.8_MZ217	1.59	3.53E-02
Sugar	Cellobiose	Cellobiose methoxime (8TMS) EZ Peak 1_ID190_RI2680.6_MZ361	3.6	2.10E-04
Sugar	Fructose	Fructose methoxime (5TMS) EZ Peak 1_ID122_RI1854.6_MZ364	10.27	6.35E-02
Sugar	Galactose	Galactose methoxime (5TMS) EZ Peak 1_ID127_RI1873.8_MZ160	16.32	5.79E-02
Sugar	Gentiobiose	Gentiobiose methoxime (8TMS) EZ Peak 1_ID199_RI2786.4_MZ361	5.79	1.28E-02
Sugar	Glucose	Glucose methoxime (5TMS)_ID128_RI1879.1_MZ160	10.36	5.88E-02
Sugar	Lyxose	Lyxose MX (4TMS) EZ Peak 1 (Major)_ID77_RI1594.4_MZ205	2.33	7.79E-02
Sugar	Maltose	Maltose methoxime (8TMS) EZ Peak 1_ID193_RI2718_MZ361	2.3	4.05E-03
Sugar	Mannose	Mannose MX (5TMS) EZ Peak 1 (Major)_ID125_RI1866.1_MZ205	4.56	2.44E-03
Sugar	Rhamnose	Rhamnose methoxime (4TMS) EZ Peak 1_ID99_RI1708.9_MZ277	3.16	5.60E-02
Sugar	Ribose	Ribose methoxime (4TMS)_ID92_RI1667.8_MZ217	1.59	3.53E-02
Sugar	Trehalose	Trehalose (8TMS)_ID194_RI2725.1_MZ191	1.81	1.00E-03
Sugar	Xylose	Xylose methoxime (4TMS) EZ Peak 2_ID89_RI1645.8_MZ217	1.38	2.36E-01
Sugar	Sucrose	Sucrose (8TMS)_ID187_RI2621_MZ451	2.1	2.00E-05
Sugar	Xylulose	[Xylulose (4TMS)_MS73_RI1565.7]_ID69_RI1565.7_MZ306	8.16	7.90E-04
Sugar	Raffinose	Unident._MST_RI3375.1_ID251_ID251_RI3375.1_MZ437	34.64	6.90E-04
Sugar	Melibiose	Unident._MST_RI2874.7_ID74_ID300_RI2874.7_MZ217	32.26	6.60E-04
Sugar Alcohol	D-Threitol	Threitol (4TMS)_ID54_RI1482.3_MZ293	3.4	2.54E-02
Sugar Alcohol	Erythritol	Erythritol (4TMS)_ID55_RI1490_MZ307	3.84	4.30E-04
Sugar Alcohol	Galactitol	Galactitol (6TMS)_ID136_RI1919.7_MZ217	3.78	2.35E-03
Sugar Alcohol	myo-Inositol	myo-Inositol (6TMS)_ID154_RI2076.6_MZ318	0.9	4.09E-01
Sugar Alcohol	Sorbitol	Sorbitol (6TMS)_ID134_RI1918.6_MZ319	6.33	1.78E-03
Sugar Alcohol	Xylitol	[Xylitol (5TMS)_MS85_RI1685.7]_ID96_RI1685.7_MZ319	1.7	7.80E-04
Sugar Alcohol	Galactinol	Unident._MST_RI2954.7_ID309_ID309_RI2954.7_MZ305	22.94	5.00E-04
Sugar Phosphate	Fructose-6-phosphate	Fructose-6-phosphate methoxime (6TMS)_ID170_RI2290.4_MZ299	1.33	3.39E-02
Sugar Phosphate	Glucose-6-phosphate	Glucose-6-phosphate methoxime (6TMS) EZ Peak 2_ID174_RI2328.1_M	1.33	4.63E-01
TCA	Citramalic acid	Citramalic acid (3TMS)_ID53_RI1462.4_MZ259	1.51	2.19E-01
TCA	Citric acid	Citric acid (4TMS)_ID117_RI1810.9_MZ465	0.45	3.30E-04
TCA	Fumaric acid	Fumaric acid (2TMS)_ID30_RI1346.7_MZ246	1.86	4.38E-02
TCA	Oxoglutaric acid	2-Ketoglutaric acid methoxime (2TMS)_ID70_RI1571.1_MZ198	0.42	5.17E-02
TCA	Succinic acid	Succinic acid (2TMS)_ID25_RI1311.9_MZ247	0.53	3.30E-04
	5-aminolevulinic acid	5-aminolevulinic acid MX (1TMS) Peak 2 (Minor)_ID113_RI1784.5_MZ34	5.44	3.77E-01
	Glycerol-2-phosphate	Glycerol-2-phosphate (4TMS)_ID101_RI1714.6_MZ299	1.53	7.68E-02
	N-Acetylserine	N-Acetyl-L-serine (2TMS)_ID56_RI1499_MZ261	1.96	8.99E-02
	O-Acetylserine	O-Acetyl-L-serine (2TMS)_ID39_RI1389_MZ216	0.91	7.78E-01

Supplementary Figure S5 - Summary of all the identified metabolites detected by GC-MS showing changes in plants subjected to 2 weeks of heat stress. Compounds were classified into chemical classes/biochemical pathways they are involved in. The name of the derivatives of the compound, the retention time, and the identifier peak was given in the third column. The values in the fourth column are the fold changes of the metabolite when comparing Heat over Control. In the fifth column, p-values as calculated by Student's T-tests (two tails, equal variance) are highlighted red if less than 0.05. (n=5).

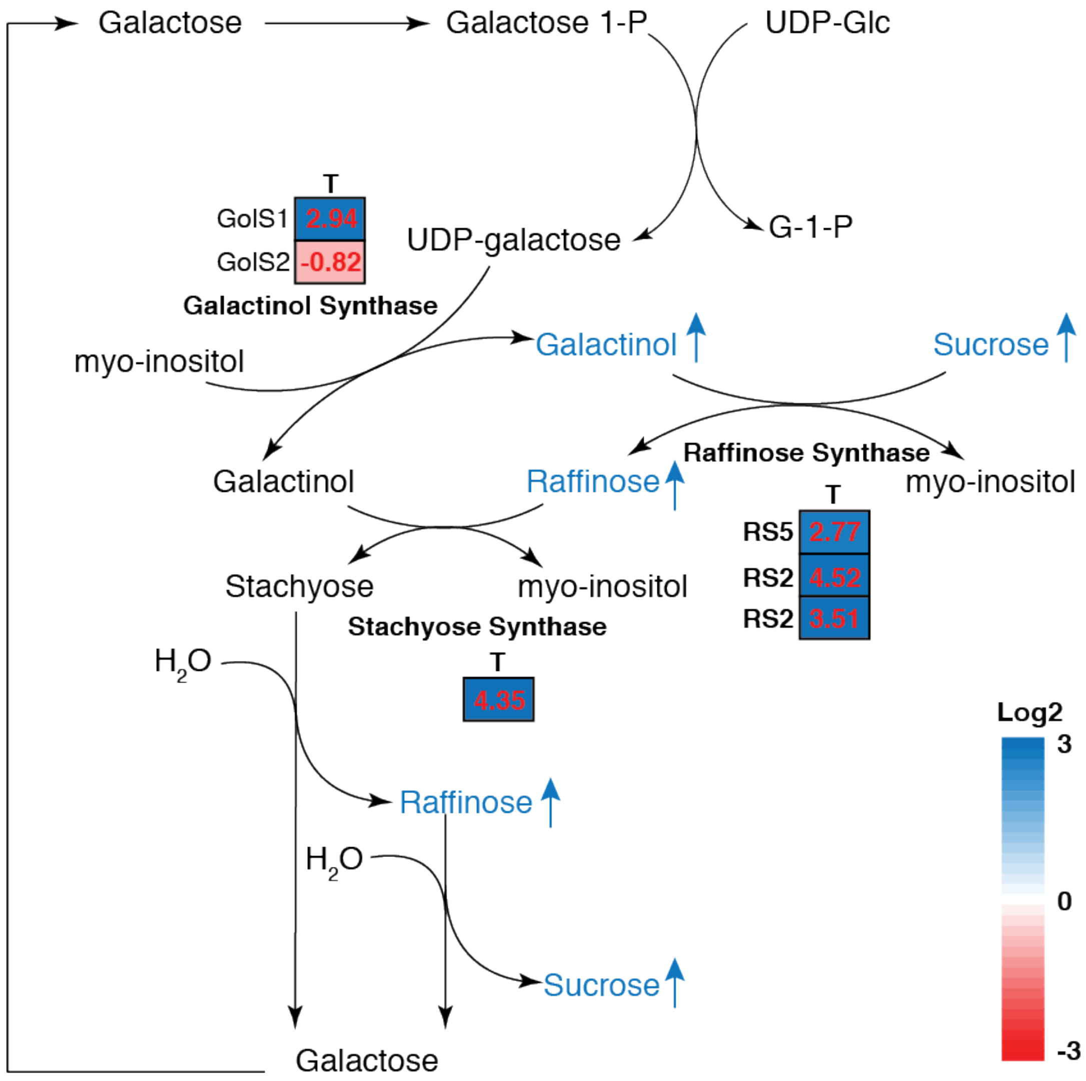

Supplementary Figure S6: Changes in the raffinose pathway induced by heat treatment. For each gene, the transcript and protein fold-changes (Heat Vs Control) in log2 scale are shown in coloured boxes, where “T” designate the change in transcript level and “P” designate protein level. The colour scale can be found on the bottom right corner. Numbers in red (or yellow if background is dark red) indicate the fold-changes are significant with $p < 0.05$. Metabolites levels which increase significantly are shown in red and metabolites levels which decrease significantly are shown in blue ($p<0.05$).

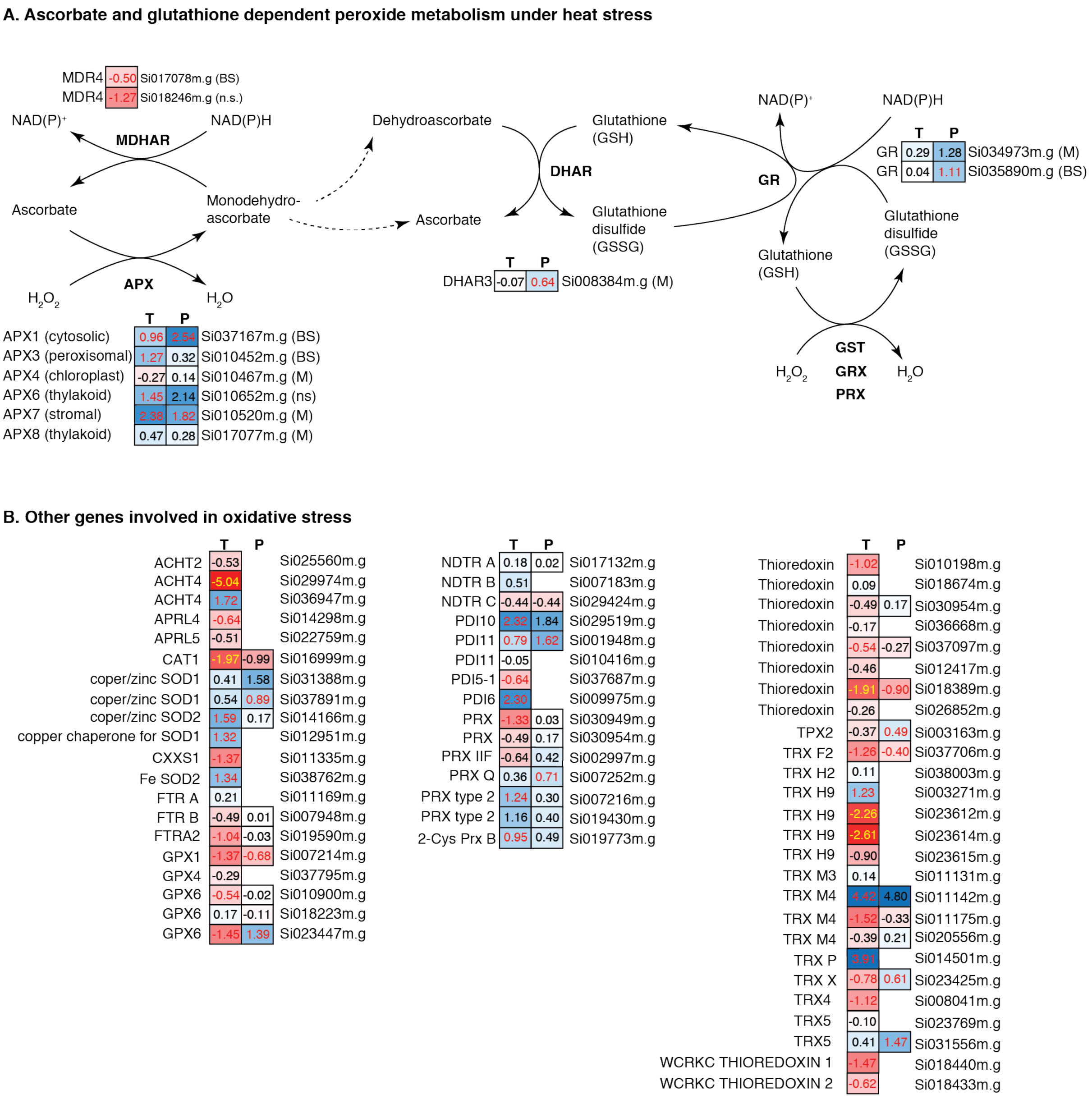

Supplementary Figure S7: Response of genes involved in oxidative stress to long-term heat stress in *S. viridis*. (A) the ascorbate-glutathione pathway and (B) other genes involved in oxidative stress response. For each gene, the transcript and protein foldchanges (Heat Vs Control) in log2 scale are shown in colored boxes, where “T” designates the change in transcript level and “P” designates protein level. The colour scale can be found on the top right corner. The numbers are in red colour when the change is significant ($p<0.05$), non-significant fold changes are in black. “N/A” indicates the protein was not detected for that gene. Abbreviations used: APX, ascorbate peroxidase; MDHAR, monodehydroascorbate reductase; DHAR, dehydroascorbate reductase; GR, glutathione reductase; GST, glutathione transferase; GRX, glutaredoxin; PRX, peroxiredoxin; GPX, glutathione peroxidase; TRX, thioredoxin.

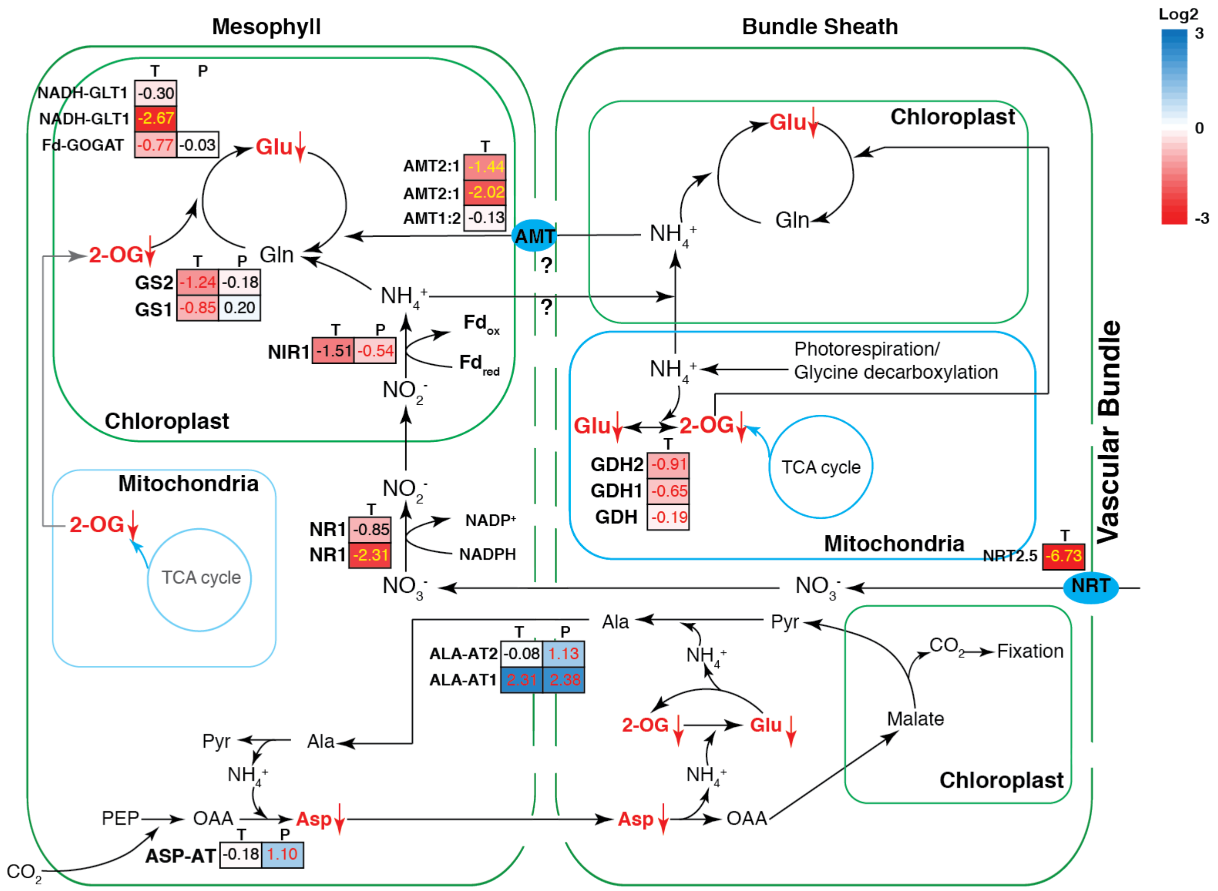

Supplementary Figure S8: Changes associated with genes encoding for proteins involved in nitrogen
assimilation and metabolism under heat stress. For each gene, the transcript and protein fold-changes (Heat Vs Control) in log2 scale are shown in coloured boxes, where “T” designate the change in transcript level and “P” designate protein level. The colour scale can be found on the top left corner. Numbers in red (or yellow if background is dark red) indicate the fold-changes are significant with $p < 0.05$. Metabolites levels which increase significantly are shown in red and metabolites levels which decrease significantly are shown in blue ($p<0.05$). Abbreviations: NADH-GLT, NADH-dependent glutamate synthase; Fd-GOGAT, ferredoxin-dependent glutamate synthase; GS, glutamine synthase; NIR, nitrite reductase; NR, nitrate reductase; AMT, ammonium transporter; ALA-AT, alanine aminotransferase; ASP-AT, aspartate amino transferase; GDH, Glutamate Dehydrogenase; NRT, nitrate transporter.

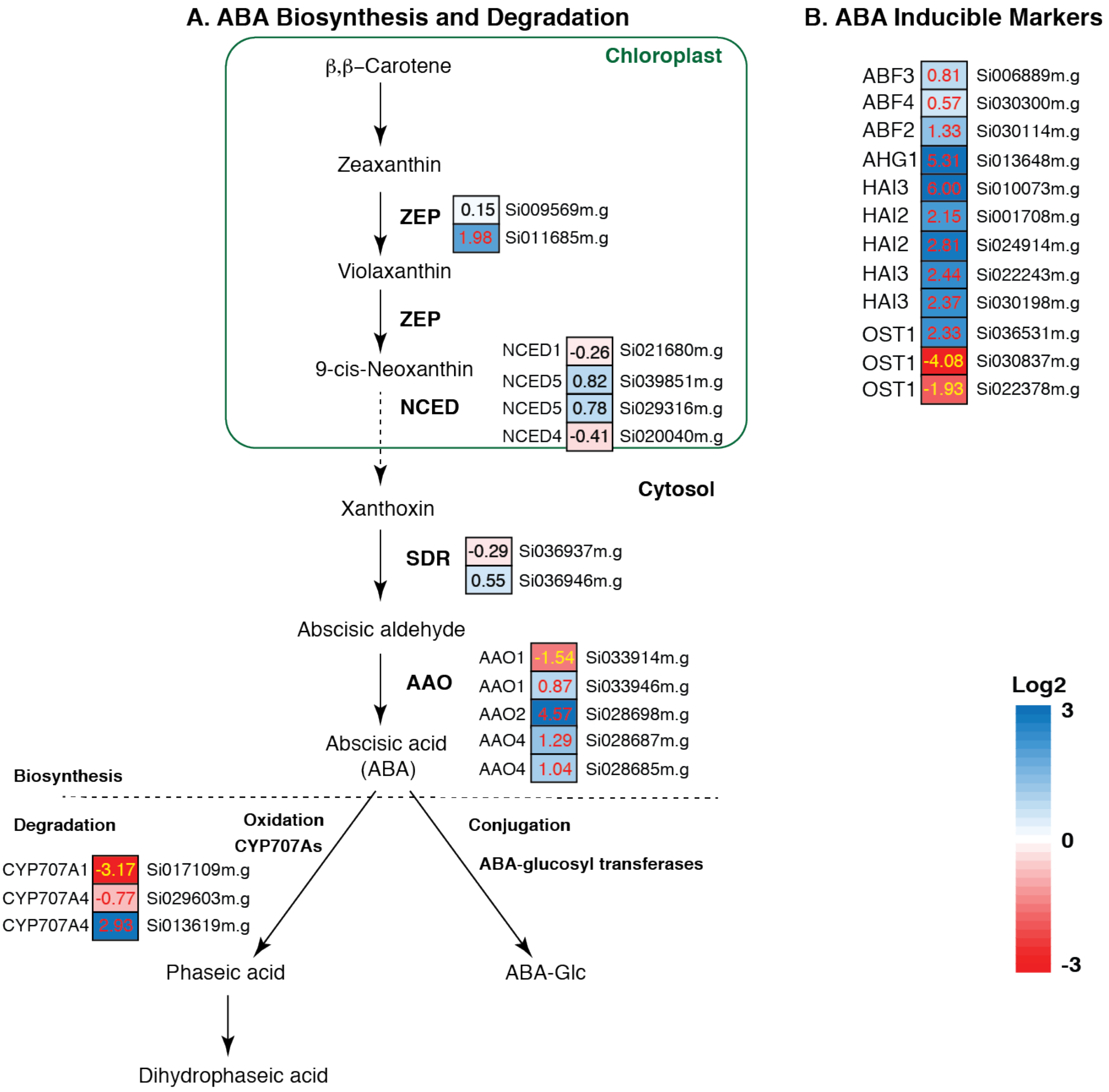

Supplementary Figure S9: Abscisic acid (ABA) transcriptional response pathways. (A) Expression
changes of genes in the ABA biosynthesis and degradation pathways. (B) Expression changes of representative ABA inducible genes. The boxes are coloured based on the log2 fold-change of the transcript expression level in the heat-stressed plants as compared to the control. The colour scale is shown on the bottom right. The numbers shown are the log2 fold change value, and are coloured red/yellow if the change is significant (with a padj value <0.05).

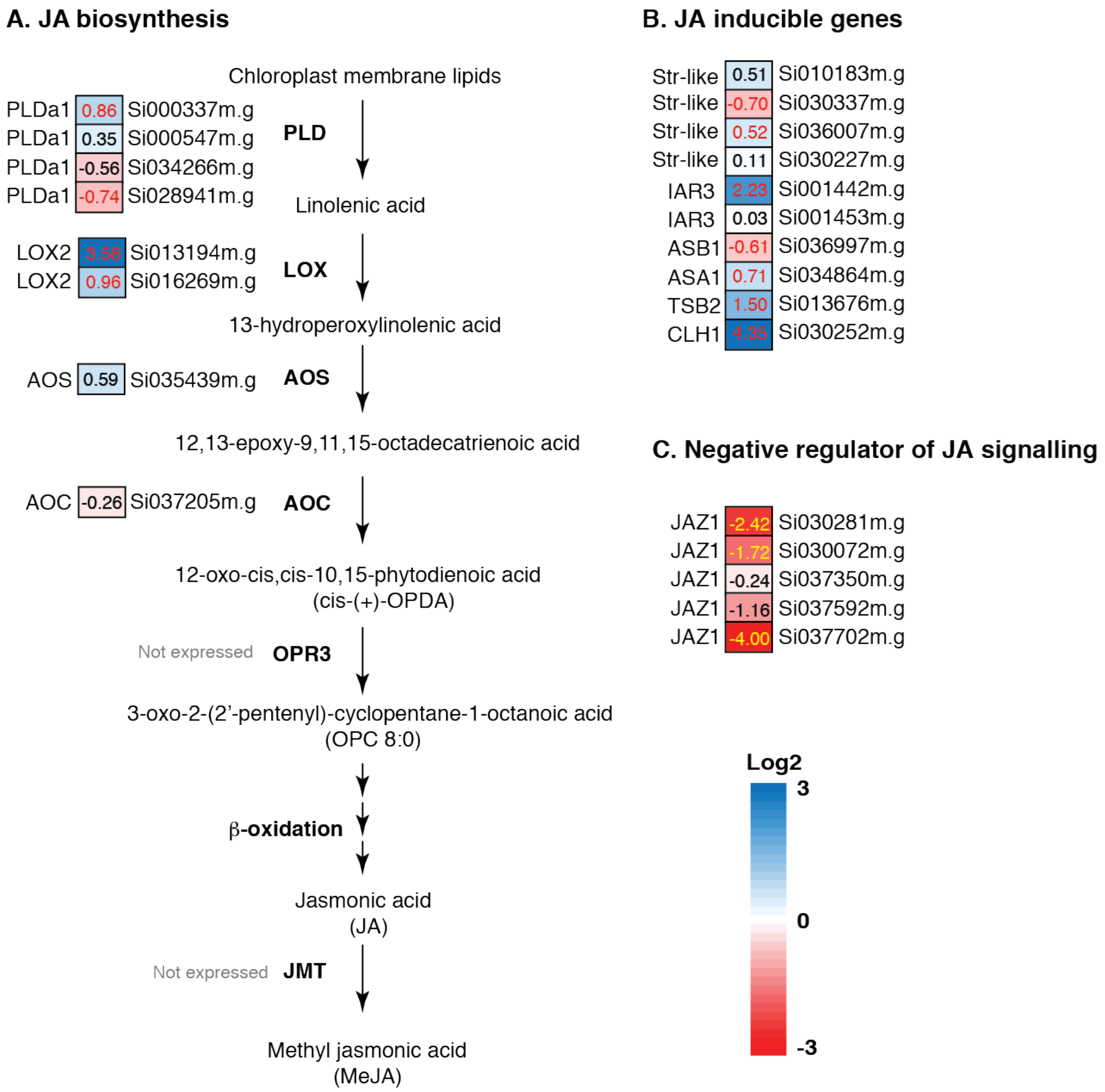

Supplementary Figure S10: Jasmonic acid (JA) transcriptional responses. (A) The expression levels of genes encoding for proteins involved in JA biosynthesis under heat stress. (B) Expression of known JA-regulated genes under heat stress. The boxes are coloured based on the log2 fold-change of the transcript expression level in the heat stressed plants as compared to the control. The colour scale is shown on the bottom right. The numbers shown are the log2 fold change value, and are coloured red/yellow if the change is significant (with a padj value <0.05).

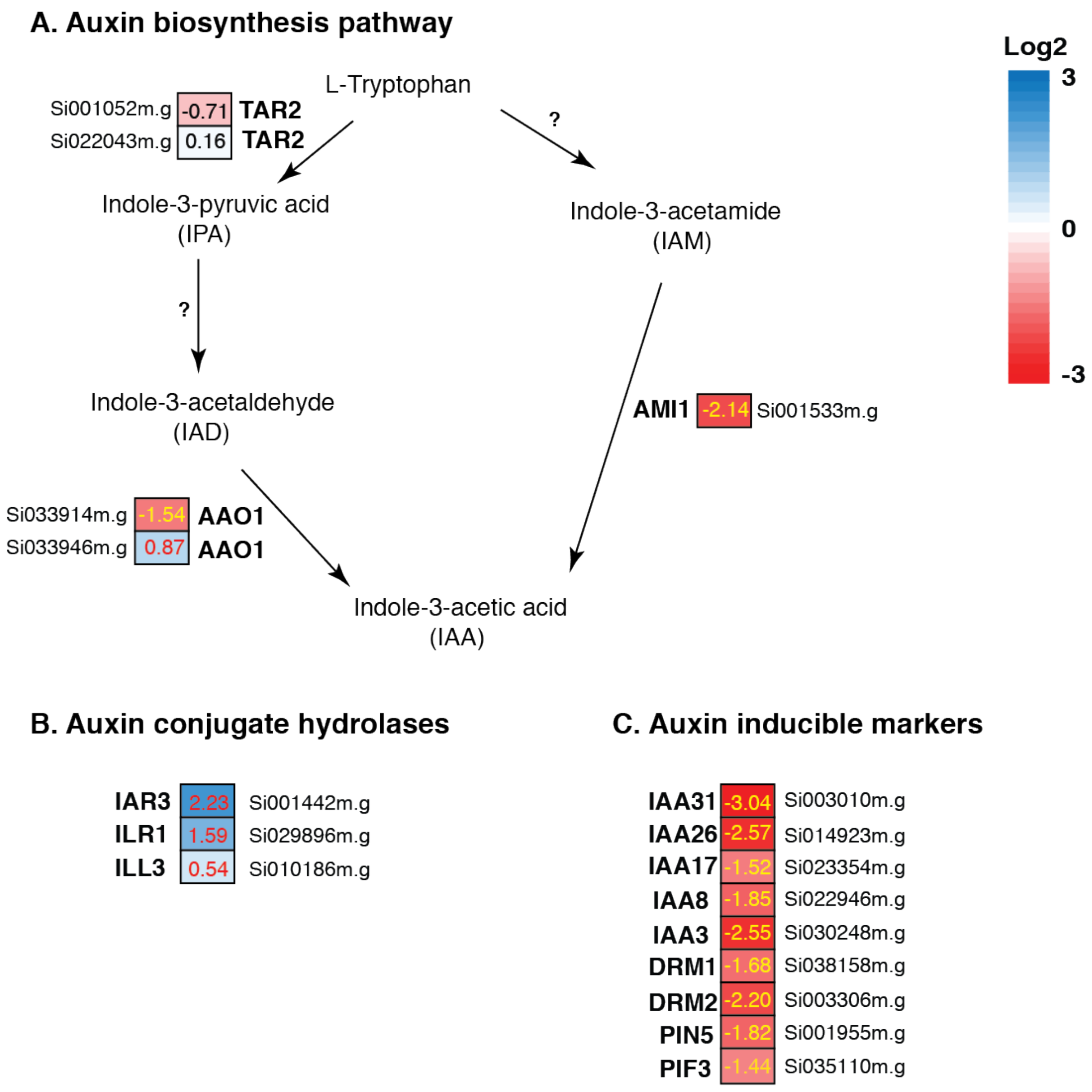

Supplementary Figure S11: Analysis of gene expression in auxin biosynthesis and degradation. Changes in transcript expression in (A) auxin biosynthesis pathway, (B) auxin degradation pathway, and (C) auxin inducible markers. The boxes are coloured based on the log2 fold-change of the transcript expression level in the heat-stressed plants as compared to the control. The colour scale is shown on the top right corner. The numbers shown are the log2 fold change value, and are coloured red/yellow if the change is significant (with a padj value <0.05). For auxin biosynthesis genes in (A), all of the genes identified and expressed in *S. viridis* are shown. For (B) and (C), only genes that were expressed in the leaves, and have changed significantly under heat are shown.

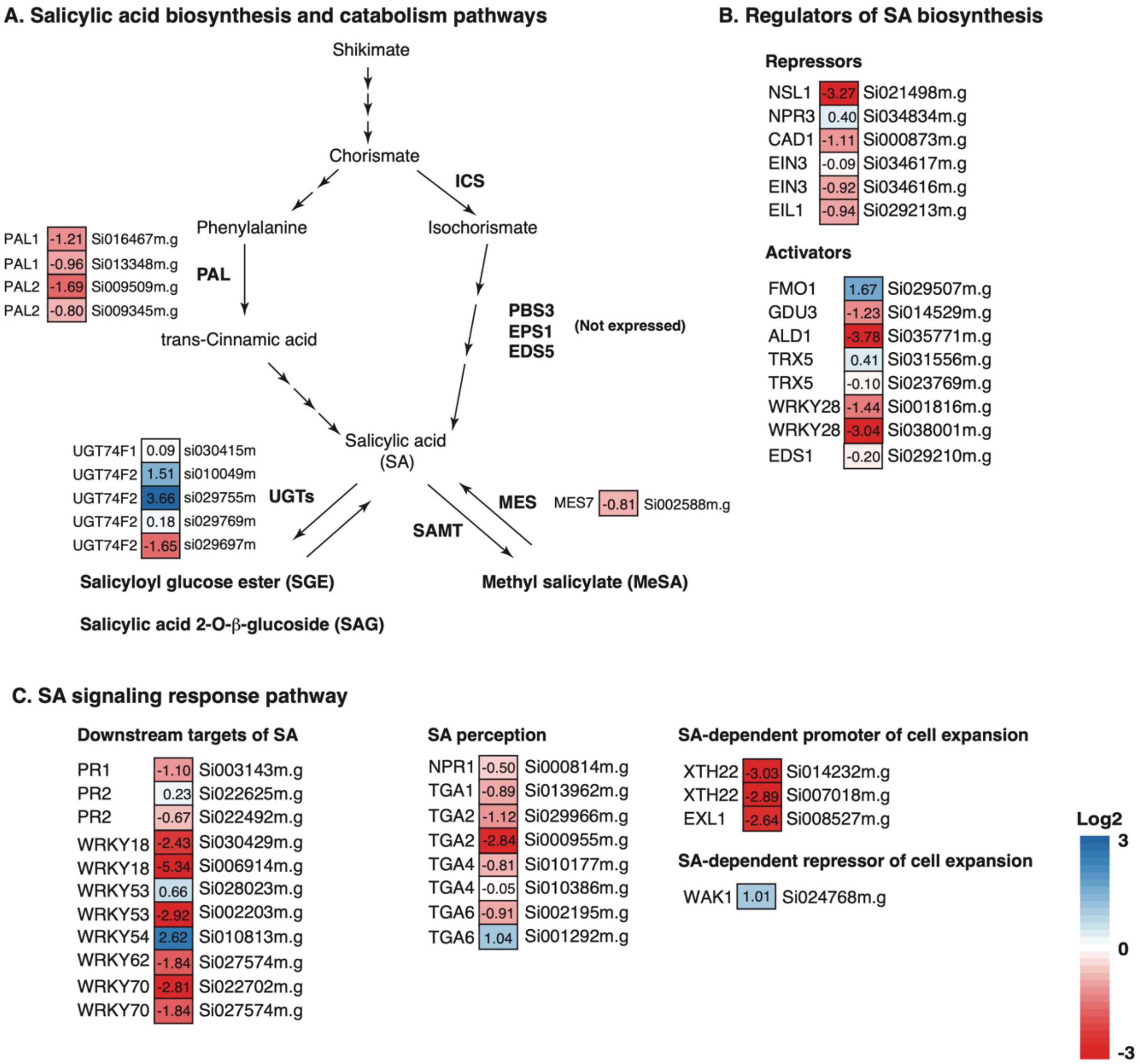

Supplementary Figure S12: Transcriptional changes associated with salicylic acid (SA) response pathway under heat stress in *S. viridis*. (A) Genes encoding for proteins involved in SA biosynthesis and catabolism. Multiple arrows indicate pathway that have not been fully elucidated, therefore genes involved are not completely known. (B) Known regulators of SA biosynthesis. (C) Genes known to be involved in SA perception, downstream targets of SA, and targets of SA that are involved in regulation of cell expansion. The boxes are coloured based on the log2 fold-change of the transcript expression level in the heat-stressed plants as compared to the control. The colour scale is shown on the bottom right. The numbers shown are the log2 fold change value.

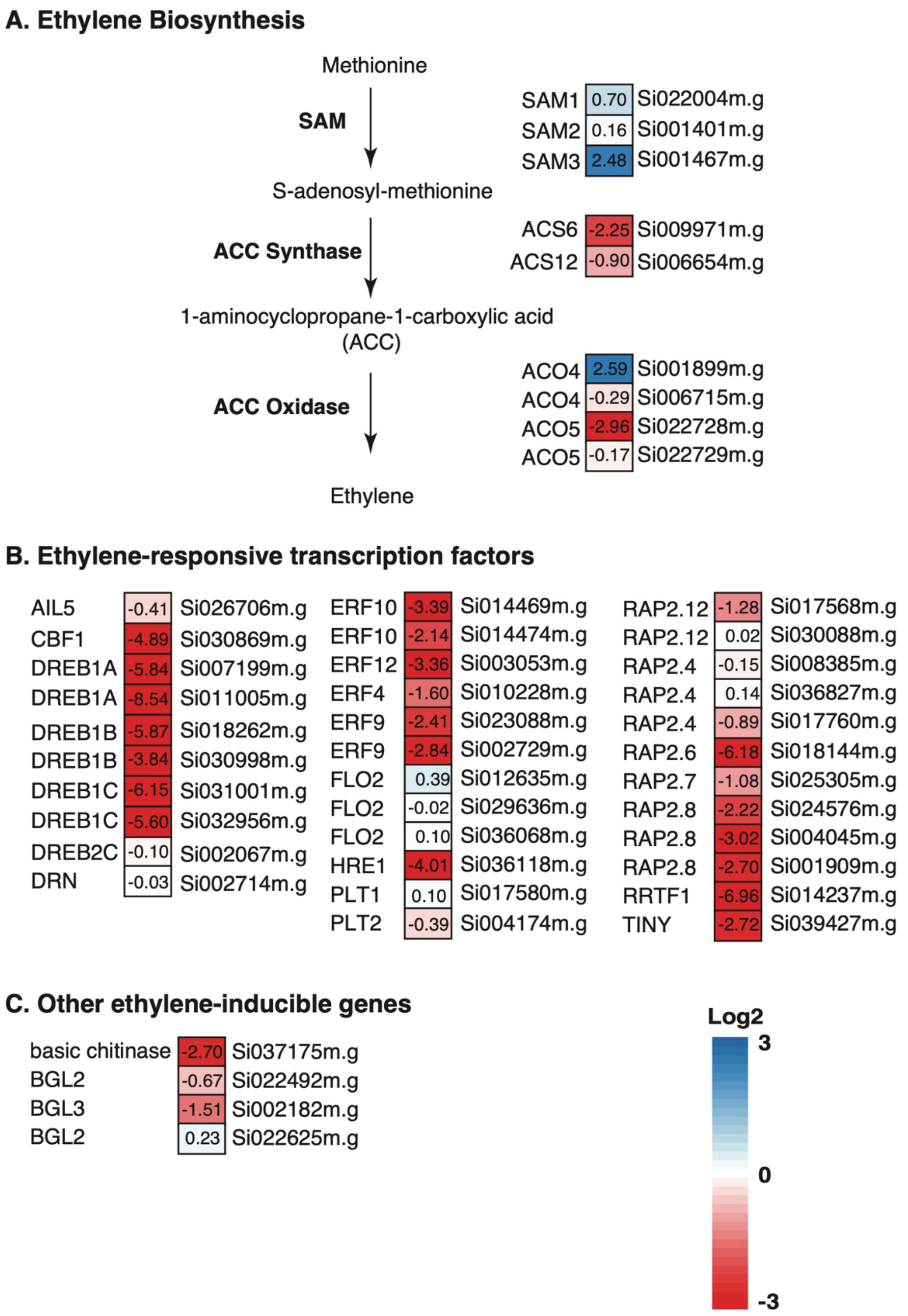

Supplementary Figure S13: The ethylene-related transcriptional responses. (A) The expression changes of genes encoding for proteins involved in ethylene biosynthesis under heat stress. (B) Expression changes of ethylene-responsive transcription factors under heat stress. (C) Other known ethylene-inducible genes. The boxes are coloured based on the log2 fold-change of the transcript expression level in the heat-stressed plants as compared to the control. The colour scale is shown on the bottom right.

A. CK biosynthesis and metabolism

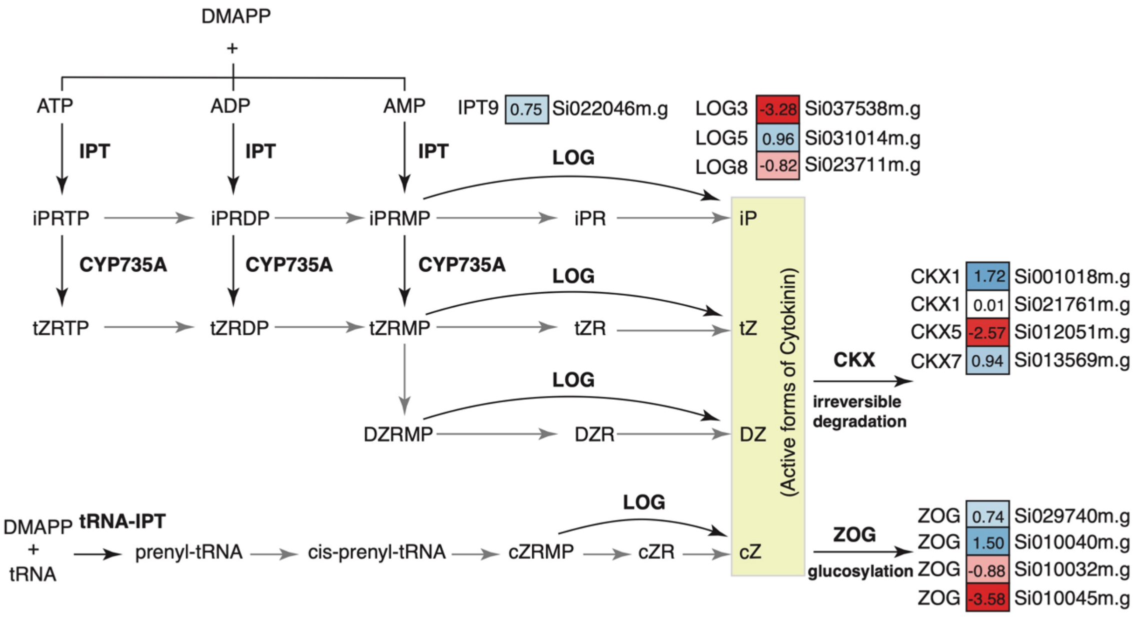

B. CK induced genes

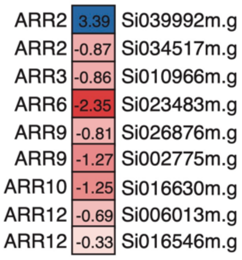

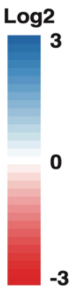

C. Setaria gene orthologs involved in CK biosynthesis and metabolism

Gene Name	ID	Gene Name	ID
IPT1	Si024711m.g	CKX1	Si021761m.g
IPT1	Si027625m.g	CKX1	Si001018m.g
IPT2	Si001384m.g	CKX4	Si019752m.g
IPT2	Si001406m.g	CKX4	Si001019m.g
IPT3	Si012132m.g	CKX5	Si001552m.g
IPT3	Si032044m.g	CKX5	Si019278m.g
IPT3	Si020675m.g	CKX5	Si008391m.g
IPT5	Si039092m.g	CKX5	Si001500m.g
IPT7	Si004945m.g	CKX5	Si000960m.g
IPT8	Si019045m.g	CKX5	Si012051m.g
IPT9	Si022046m.g	CKX6	Si035175m.g
CYP735A1	Si015770m.g	CKX7	Si013710m.g
CYP735A2	Si032720m.g	CKX7	Si013569m.g
LOG1	Si039293m.g	ZOG	Si029740m.g
LOG1	Si012782m.g	ZOG	Si010040m.g
LOG3	Si039738m.g	ZOG	Si010038m.g
LOG3	Si037538m.g	ZOG	Si012663m.g
LOG4	Si002703m.g	ZOG	Si010032m.g
LOG5	Si031014m.g	ZOG	Si012135m.g
LOG6	Si002934m.g	ZOG	Si010045m.g
LOG8	Si023711m.g	N-GT	Si032090m.g
		N-GT	Si032122m.g
		N-GT	Si032401m.g

Supplementary Figure S14: Transcriptional response associated with cytokinins (CKs). (A) CK biosynthesis and metabolism pathway, (B) CK inducible genes, and (C) identified orthologues of genes involved in CK biosynthesis and metabolism in *Setaria*. Numbers in (A) and (B) are shown in Log2 scale. Boxes are coloured to indicate up- or down-regulation, and the color scale can be found on the bottom left corner.

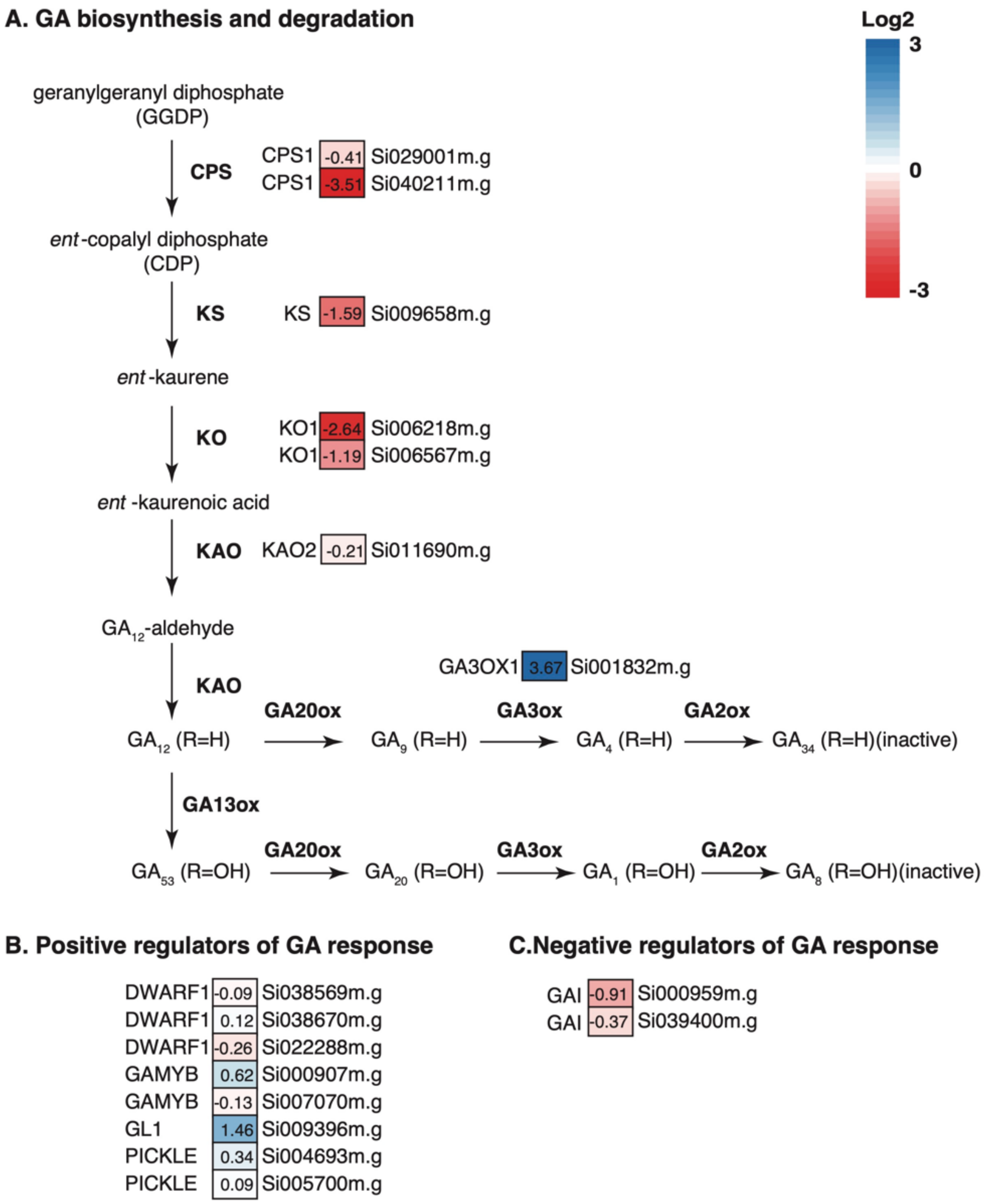

Supplementary Figure S15: The gibberellic acid (GA) transcriptional response under heat. (A) GA biosynthesis and degradation pathway. (B) Positive regulators of GA response. (C) Negative regulators of GA response. Numbers shown are in Log2 scale. Boxes are coloured to indicate up- or down-regulation, and the color scale can be found on the top right corner.

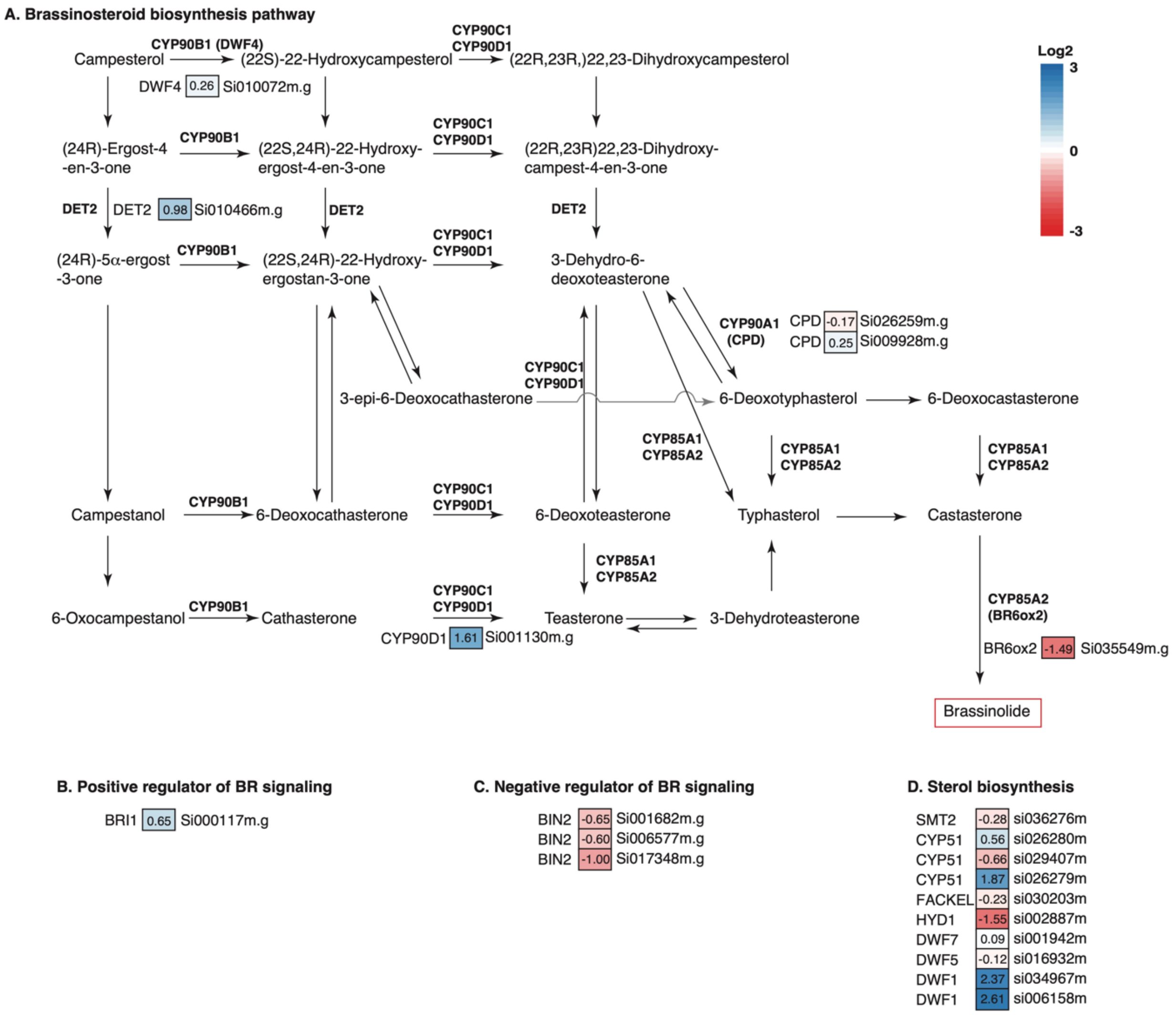

Supplementary Figure S16: The transcriptional response associated with brassinosteroids (BR) in the heat-stressed S.viridis. (A) The BR biosynthesis pathway and genes involved in BR biosynthesis. (B) Positive regulator of BR signaling. (C) Negative regulator of BR signaling. (D) Genes involved in the synthesis of campesterol. Numbers shown are in Log2 scale. Boxes are colored to indicate up- or down-regulation, and the color scale can be found on the top right corner.

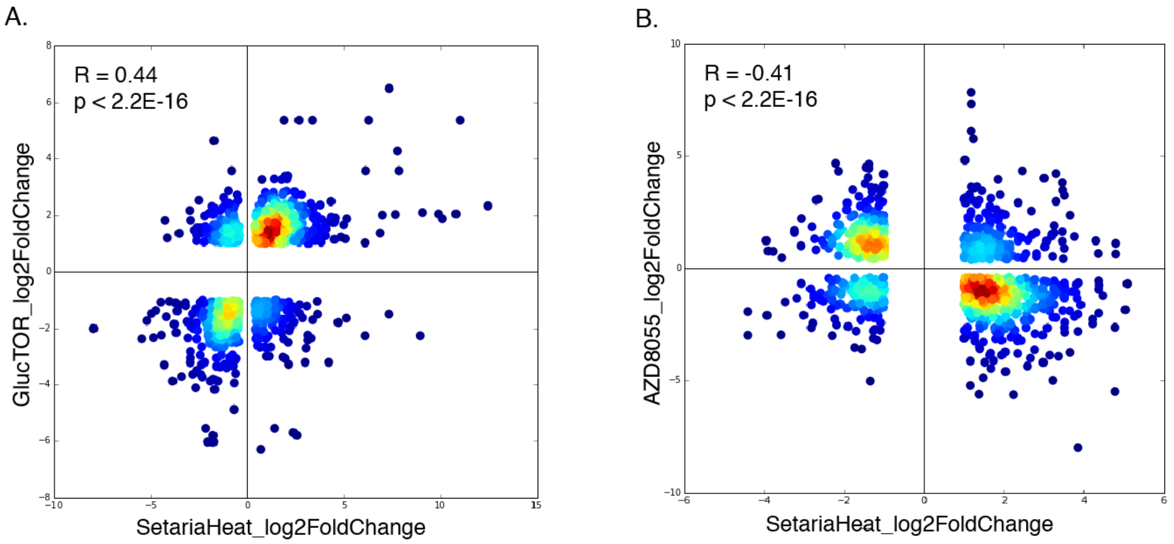

Supplementary Figure S17. Cross-species correlation analysis between transcriptional responses of *Arabidopsis thaliana* TOR-regulated genes and those of their *Setaria viridis* orthologs: *S. viridis* prolonged heat stress vs (A) *A. thaliana* TOR-mediated glucose activated genes (Xiong et al., 2013); (B) *A. thaliana* response to TOR-inhibitor chemical, AZD8055 (Dong et al., 2015). The *S. viridis* orthologs of Arabidopsis TOR-regulated genes were identified by mapping orthologues from Arabidopsis to Setaria using the orthologue mappings contained in the gene annotation file 'Sitalica_312_v2.2.annotation_info.txt' associated with version 2.2 of the *S. italica* reference genome (Bennetzen et al., 2012) available from the Phytozome website (Goodstein et al., 2012). Transcript fold-changes were $\log_2$-transformed and plotted against each other using a density scatter plot function in the 'matplotlib' Python package. The data points are coloured according to the density of data points with dark blue indicating the lowest density and red the highest density. Pearson correlation coefficient and p-value for each association are displayed on each plot.

Supplementary Methods

Gene Ontology (GO) enrichment analysis

The list of gene IDs for significantly up- and down-regulated genes (padj < 0.05) in the heat-stressed plants were submitted to the agriGO website (bioinfo.cau.edu.cn/agriGO) for functional characterization using its singular enrichment analysis function. The *Setaria italica* v2.1 reference genome database (Bennetzen et al., 2012) available from the Phytozome website (Goodstein et al., 2012)was used to assign GO terms to genes. Only the list of expressed genes (mean RPKM > 20 in both of the conditions) was used as the reference background when considering enrichment. A GO category was considered significantly over-represented when the hypergeometric test p < 0.05, and a Benjamini-Yekutieli FDR < 0.1, and at least five entries mapped. The full listing of GO terms can be found in Supplementary Table 3 (for up-regulated genes) and Supplementary Data File 1 - Table 4 (for down-regulated genes). To reduce the complexity of the GO dataset, the list of significant GO terms in the Biological process category was input into REViGO (Supek et al., 2011) to remove functional and semantic redundancies. The simplified list of up-regulated GO terms output by REViGO is provided in Supplementary Data File 1 - Table 5.

References

Bennetzen, J.L., Schmutz, J., Wang, H., Percifield, R., Hawkins, J., Pontaroli, A.C., Estep, M., Feng, L., Vaughn, J.N., Grimwood, J., Jenkins, J., Barry, K., Lindquist, E., Hellsten, U., Deshpande, S., Wang, X., Wu, X., Mitros, T., Triplett, J., Yang, X., Ye, C.-Y., Mauro-Herrera, M., Wang, L., Li, P., Sharma, M., Sharma, R., Ronald, P.C., Panaud, O., Kellogg, E.A., Brutnell, T.P., Doust, A.N., Tuskan, G.A., Rokhsar, D., and Devos, K.M. (2012). Reference genome sequence of the model plant Setaria. Nature Biotechnology **30,** 555-561.

Cousins, A.B., Badger, M.R., and von Caemmerer, S. (2006). Carbonic anhydrase and its influence on carbon isotope discrimination during C4 photosynthesis. Insights from antisense RNA in Flaveria bidentis. Plant physiology **141,** 232-242.

Dong, P., Xiong, F., Que, Y., Wang, K., Yu, L., Li, Z., and Maozhi, R. (2015). Expression profiling and functional analysis reveals that TOR is a key player in regulating photosynthesis and phytohormone signaling pathways in Arabidopsis. Frontiers in Plant Science **6**.

Evans, J.R., Sharkey, T.D., Berry, J.A., and Farquhar, G.D. (1986). Carbon Isotope Discrimination measured Concurrently with Gas Exchange to Investigate CO₂ Diffusion in Leaves of Higher Plants. Functional Plant Biology **13,** 281-292.

Goodstein, D.M., Shu, S., Howson, R., Neupane, R., Hayes, R.D., Fazo, J., Mitros, T., Dirks, W., Hellsten, U., Putnam, N., and Rokhsar, D.S. (2012). Phytozome: a comparative platform for green plant genomics. Nucleic Acids Research **40,** D1178-D1186.

Henderson, S.A., von Caemmerer, S., and Farquhar, G. (1992). Short-Term Measurements of Carbon Isotope Discrimination in Several C4 Species.

Pengelly, J.J., Sirault, X.R., Tazoe, Y., Evans, J.R., Furbank, R.T., and von Caemmerer, S. (2010). Growth of the C4 dicot Flaveria bidentis: photosynthetic acclimation to low light through shifts in leaf anatomy and biochemistry. Journal of experimental botany **61,** 4109-4122.

Supek, F., Bošnjak, M., Škunca, N., and Šmuc, T. (2011). REVIGO Summarizes and Visualizes Long Lists of Gene Ontology Terms. PLOS ONE **6,** e21800.

Tazoe, Y., von Caemmerer, S., Badger, M.R., and Evans, J.R. (2009). Light and CO2 do not affect the mesophyll conductance to CO2 diffusion in wheat leaves. Journal of Experimental Botany **60,** 2291-2301.

Xiong, Y., McCormack, M., Li, L., Hall, Q., Xiang, C., and Sheen, J. (2013). Glucose–TOR signalling reprograms the transcriptome and activates meristems. Nature **496,** 181-186.
